# A Cycle Slip Repair Method Against Ionospheric Effects and Observational Noises for BDS Triple-Frequency Undifferenced Phases

**DOI:** 10.3390/s20102819

**Published:** 2020-05-15

**Authors:** Dehai Li, Jinzhong Mi, Pengfei Cheng, Yunbin Yuan, Xingli Gan

**Affiliations:** 1 Chinese Academy of Surveying and Mapping, No.28, Lianhuachi West Road, Beijing 100830, China; lidh@casm.ac.cn (D.L.); goldheal@casm.ac.cn (J.M.); 2 Innovation Academy for Precision Measurement Science and Technology, Chinese Academy of Science, Xiaohongshan West Road, Wuhan 430071, China; yybgps@asch.whigg.ac.cn; 3 State Key Laboratory of Satellite Navigation System and Equipment Technology, Shijiazhuang 050081, China; ganxingli@163.com

**Keywords:** BDS, ionospheric variation, triple-frequency combinations, optimized combinations, cycle slip repair, cycle slip detection

## Abstract

The cycle slip detection (CSD) and cycle slip repair (CSR) are easily affected by ionospheric delay and observational noise. Aiming at mitigating the above disadvantage, a new BeiDou navigation satellite system (BDS) triple-frequency CSR method (BTCSR) is proposed for the undifferenced phase. BTCSR learns from the classic triple-frequency CSR (CTCSR), with combinations of phases and pseudoranges in correcting ionospheric delay and optimizing observational noise. Different from CTCSR, though, BTCSR has made the following improvements: (1) An optimal model of calculating cycle slip combination is established, which further takes into account the minimization of the effect of residual ionospheric error after the correction. The calculation of cycle slip combination is obtained with the root mean squared errors (0.0646, 0.1261, 0.1069) of cycles, resulting in CSR success rate of 99.9927%, and the wavelengths (4.8842,3.5738,8.1403) of m. (2) A discriminant function is added to guarantee the CSR correctness. This function utilizes epoch-difference value of the ionosphere-free and geometry-free phase to select the correct cycle slip value, which eliminates the interference of large pseudorange errors in determining the final cycle slip. Consequently, the performances of BTCSR and CTCSR have been compared. For the real BDS pseudorange observation with additional 1.5 m errors, which can cover situations of 99.96% pseudorange noise, results of CTCSR show failure, but results of BTCSR keep correct. Moreover, BTCSR has made the following improvements relative to the geometry-free cycle slip detection method (GFCSD) and Melboune–Wubbena cycle slip combination detection method (MWCSD): (1) During a moderate magnetic storm of level 6, CSR testing, with the BDS monitoring station in a low latitude region, showed that some failures occur in GFCSD because of severe ionospheric variation, but BTCSR could correctly identify and fix cycle slips. (2) For the BDS observation data with an additional 1.5 m error on the actual pseudoranges, MWCSD exhibited failures, but the repair results of BTCSR were correct and reliable. (3) For the special slips of (0,59,62) cycles, and equal slips of (1,1,1) cycles on (B1,B2,B3), that are hard to detect by GFCSD and MWCSD, respectively, BTCSR could repair these correctly. Finally, BTCSR obtains reliable repair results under large pseudorange errors and severe ionospheric variations, and the cut-off elevation larger than 10 degrees is the suggested background.

## 1. Introduction

Since BeiDou navigation satellite system (BDS) constellations have completed global coverage, BDS are providing global services. The services include the applications of navigation, positioning, and timing, where all precise services utilize the phase data. Continuous phase data without cycle slip ensures stable and precise results for real time kinematic positioning and precise point positioning [1,2,3,4,5]. However, the phase observations are often interrupted due to the influence of the observation environment, especially when the BDS signal is blocked by buildings, trees, and so on. At this moment, the cycle slip appears in phase data. Therefore, the detection and repair of cycle slip become the basic procedure of global navigation satellite system (GNSS) data processing, and it is the primary task for precise positioning and timing with the phase data [6,7,8,9,10].

Many effective cycle slip detection (CSD) methods have been provided. Blewitt [11] and Bisnath [12] proposed CSD methods with the Melboune–Wubbena (MW) combination [13] and the geometry-free phase. However, the geometry-free phase is still affected by ionospheric delay, which may destroy the correctness of CSD results. MW combination excludes the ionospheric delay but includes the pseudorange error. The large pseudorange errors easily occurring in observations can cause significant deviation in MW combination, which even leads to the misjudgment of CSD, and seriously degrades the reliability of CSD. In the triple-frequency case, cycle slips of MW combinations cannot be directly transferred to the basic cycle slips. Thus the MW combinations are unable to independently repair the cycle slip on the basic carriers. 

Outlier filtering and cycle slip estimation are taken to the cycle slip repair (CSR). Kim and Langley [14] introduced the generating and filtering of cycle slip candidates. Ji et al. [15] designed Single-epoch ambiguity resolution with the Least-squares Ambiguity Decorrelation Adjustment (LAMBDA) technique [16]. Zhang et al. [17] handled the cycle slip with the reinitialization of ambiguity. At present, CSD with geometry-free phase and the ambiguity reinitialization method are used to process cycle slips in mature GNSS software, and reliable results have been obtained.

Cycle slip estimation and correction in epoch-difference mode can fulfill the CSR [18,19]. Using the epoch-difference data of multiple observable satellites, cycle slips can be estimated from the rigorous observation model, while CSR for a single satellite’s data needs to find other methods. Outlier detection and cycle slip prediction are employed to realize CSR, but its repair effect depends on the prediction accuracy [20]. However, the accuracy of cycle slip prediction is limited by the sampling interval, the observational error, and the dynamic characteristics of the carrier. CSR, with a hybrid of MW combination and geometry-free phase, achieves good posterior performance [21]. The ionospheric variation is corrected well from the geometry-free phase by smoothing algorithm [9,22,23]. The accuracy of the MW combination calculation is improved in the smoothing mode as well. While the smoothing mode requires that the observations within the smoothing window are without cycle slip, for real-time processing, it is difficult to meet the requirement of window smoothing on the current and subsequent observations. In order to fulfill real-time CSR of single satellite data, researchers have turned their ideas to the combinations of triple-frequency phases and pseudoranges, and their epoch-difference application in CSR [1,24,25,26].

Using the combinations of BDS triple-frequency phases and pseudoranges, many studies have been carried out on CSR. The conventional idea is the following [22,27,28,29,30]. Firstly, the combinations with triple-frequency phases and pseudoranges are calculated. Secondly, the ionospheric correction should be carried out. Since the ionosphere is active, it changes dramatically during the magnetic storm [1,31,32,33]. The ionospheric variation will be amplified by the combination, and will give rise to a striking deviation of more than 0.5 cycles on the cycle slip combination [23,30,34]. Thirdly, the epoch-difference operation is implemented on the combinations, and floating values of the combined cycle slips are obtained and fixed. At last, the combined cycle slips are converted to the basic cycle slips on the triple-frequency carriers by the integer transformation. 

However, the influence of residual ionospheric error after correction still needs to be comprehensively optimized according to the requirements of cycle slip calculation. On the other hand, the large pseudorange error can still produce a significant deviation more than 0.5 cycles on the combined cycle slip calculation, even though the combination noise has been optimized. Significant deviations over 0.5 cycles will cause the failure of fixing the combined cycle slip, and result in the failure of repairing the basic cycle slip too. Therefore, overcoming the CSR failures caused by large observation noise and severe ionospheric variation is still a key issue for real-time single-satellite CSR.

By learning from the classic triple-frequency CSR with the combinations of phases and pseudoranges (CTCSR), this study develops a BDS triple-frequency CSR method (BTCSR). The BTCSR references the conventional idea from CTCSR, and the similarities between BTCSR and CTCSR are the following: (1) the variation of ionospheric delay is corrected in the combination of phases and pseudoranges; and (2) the optimization of observational noises is conducted to calculate the combined cycle slip. On the other hand, BTCSR makes some little improvements to the CTCSR. The differences between BTCSR and CTCSR are the following: (1) the optimization of calculating cycle slip combination further takes into account the influence of residual ionospheric error after the correction, and the effect of residual ionospheric error on the combined cycle slip calculation is minimized too; and (2) a discriminant function is formed to select the correct cycle slip. This function sorts the absolute epoch-difference value of the ionosphere-free and geometry-free phase to determine the final cycle slip. The interferences of larger pseudorange error and ionospheric delay are thereby eliminated in the process of determining the correct cycle slip. 

The performance of BTCSR is validated with real BDS triple-frequency data. The improvements of BTCSR are investigated by comparison to the CTCSR, the traditional CSD with the geometry-free phase (GFCSD), and the CSD with MW combination (MWCSD). In the following, the mathematical model of BTCSR is established. In the meantime, the effect of the ionospheric correction, the contribution of the optimal cycle slip calculation, and the feasibility of pseudorange error elimination, are validated carefully. A subsequent section introduces the testing schemes and processing strategies. In the next section, experimental results of the improved method with real data are presented. At last, some beneficial conclusions are summarized.

It should be noted that the values of the combination coefficients supplied by this study are only suitable to the BDS, because the optimization is implemented on the basis of the BDS frequencies. In BDS CSR, the comparisons are investigated between the BTCSR, the conventional CSR with BDS optimized combinations, the CSR with BDS Melboune–Wubbena combinations, and the BDS geometry-free phase. The comparison of BDS and the other GNSS may be another task, and is not focused on in this paper. For the other GNSS, the frequencies, signal and measurement quality, precise orbit and clock, and precise positioning performance, have been comprehensively compared and concisely illustrated among the BDS, quasi-zenith satellite system and GPS [35].

## 2. Method

### 2.1. Cycle Slip Presentation and Analyzation

When BDS signal is lost but recaptured in an observation interval, the ambiguity of carrier phase has changed, and a cycle slip occurs:

(1) $$\Delta N_{j}=N_{j,k}-N_{j,k-1}$$

where $\Delta N_{j}$ is the cycle slip on the frequency *j* at epoch *k*; Δ is the epoch-difference operator on adjacent epochs; and $N_{j,k}$ is the ambiguity of carrier phase on the frequency *j* at epoch *k*.

If variations in the instrumental bias and multipath effect between adjacent epochs are small [11,36,37], and ionospheric delay has been corrected, combinations of phases and pseudoranges can be used to calculate the cycle slips:

(2) $$\Delta N_{j}=\frac{\Delta p_{j}}{\lambda_{j}^{}}-\Delta \phi_{j}-\frac{2\Delta {\mathrm{iono}}_{j}^{}}{\lambda_{j}^{}}$$

and the variance of cycle slip is presented:

(3) $$\sigma_{\Delta N_{j}^{}}^{2}=\frac{\sigma_{\Delta p_{j}^{}}^{2}}{\lambda_{j}^{2}}+\sigma_{\Delta \phi_{j}^{}}^{2}+\frac{4\sigma_{j,\Delta \mathrm{iono}}^{2}}{\lambda_{j}^{2}}$$

where $\lambda_{j}^{}$ is the wavelength on the frequency *j*; $\Delta p_{j}^{}$ is the epoch-difference pseudorange on the frequency *j*; $\Delta \phi_{j}$ is the epoch-difference phase; $\Delta {\mathrm{iono}}_{j}^{}$ is the epoch-difference ionospheric delay, that is, the ionospheric variation; $\sigma_{\Delta N_{j}^{}}^{2}$ is the variance of $\Delta N_{j}$; $\sigma_{\Delta p_{j}}^{2}$ is the variance of $\Delta p_{j}^{}$; $\sigma_{\Delta \phi_{j}^{}}^{2}$ is the variance of $\Delta \phi_{j}$; and $\sigma_{j,\Delta \mathrm{iono}}^{2}$ is the variance of $\Delta {\mathrm{iono}}_{j}^{}$ with the unit of $m_{}^{2}$. Analogous to GPS observation noise levels [35,36], if the standard deviation of BDS pseudorange $\sigma_{p_{j}}^{}$ is 0.3 m, and that of the carrier phase $\sigma_{\phi_{j}}^{}$ is 0.01 cycles, then $\sigma_{\Delta p_{j}^{}}^{2}$ = 0.18 $m_{}^{2}$, $\sigma_{\Delta \phi_{j}^{}}^{2}$ = 0.0002 $cycle_{}^{2}$.

### 2.2. Ionospheric Correction and Its Precision

Ionospheric delay on frequency $f_{1}^{}$ can be calculated by triple-frequency phases:

(4) $${\mathrm{iono}}_{1}^{}=(\frac{L_{1}^{}-L_{2}^{}}{1-f_{1}^{2}/f_{2}^{2}}+\frac{L_{1}^{}-L_{3}^{}}{1-f_{1}^{2}/f_{3}^{2}})/2$$

where $L_{j}^{}=(\phi_{j}^{}{+N}_{j}^{})\lambda_{j}^{}$. The ionospheric variation between adjacent epochs is expressed as:

(5) $$\Delta {\mathrm{iono}}_{1,k}^{}={\mathrm{iono}}_{1,k}^{}-{\mathrm{iono}}_{1,k-1}^{}$$

where ${\mathrm{iono}}_{1,k}^{}$ is ionospheric delay on frequency $f_{1}^{}$ at epoch *k*. If there is no cycle slip, ionospheric variation can be simplified as:

(6) $$\Delta {\mathrm{iono}}_{1,k}^{}=(\frac{\Delta \phi_{1}^{}\lambda_{1}^{}-\Delta \phi_{2}^{}\lambda_{2}^{}}{1-f_{1}^{2}/f_{2}^{2}}+\frac{\Delta \phi_{1}^{}\lambda_{1}^{}-\Delta \phi_{3}^{}\lambda_{3}^{}}{1-f_{1}^{2}/f_{3}^{2}})/2$$

First-order difference of ionospheric delay denotes as $\Delta {\mathrm{iono}}_{1,k}^{}$, its precision is

(7) $$\delta_{1,\Delta \mathrm{iono}}^{2}=(\frac{\delta_{\Delta \phi_{1}^{}}^{2}\lambda_{1}^{2}+\delta_{\Delta \phi_{2}^{}}^{2}\lambda_{2}^{2}}{(1-f_{1}^{2}/f_{2}^{2}{)}_{}^{2}}+\frac{\delta_{\Delta \phi_{1}^{}}^{2}\lambda_{1}^{2}+\delta_{\Delta \phi_{3}^{}}^{2}\lambda_{3}^{2}}{(1-f_{1}^{2}/f_{3}^{2}{)}_{}^{2}})/4$$

Obviously, the ionospheric variation can be gotten from the differential phases. Considering the $\Delta {\mathrm{iono}}_{1,k}^{}$ can be ignored, epoch-difference of the geometry-free phase is successfully applied to CSD. The traditional CSD method with geometry-free phase has been widely used in various GNSS data processing software. Further, the applicable conditions of this method are analyzed: the product of the ionospheric variation and the ionospheric amplification factor in geometry-free phase is close to 0. That is, $0.69\Delta {\mathrm{iono}}_{1,k}^{}\to 0$, where 0.69 is the maximum ionospheric amplification factor in the geometry-free phase of triple-frequency BDS.

**Figure 1:**
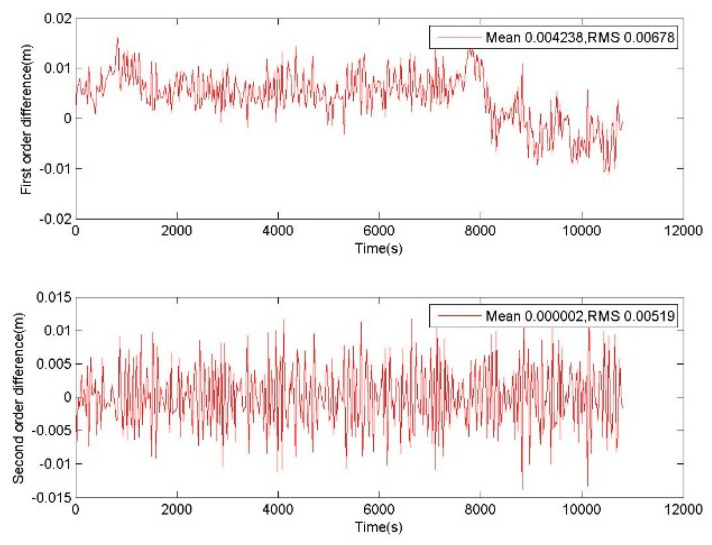
First-order and second-order differences of ionospheric delay on the satellite 1 of BeiDou navigation satellite system (BDS), whose observation data was collected by CUT0 at south latitude of 32 degrees starting from UTC 2016/05/08 00:00:00.

The ionospheric variation can be investigated via the GNSS data during the moderate magnetic storm of level 6 in the low latitude region. For an observation interval of 30 s, the first-order ionospheric difference is obtained to describe the epoch variation of ionospheric delay, as shown in Figure 1. The mean of the ionospheric variation is about 0.0042 m. This shows that the ionospheric delay has a significant effect on the epoch-difference phase. In the geometry-free phase, the ionospheric delay is reduced to 0.69 times, so it is considered that the influence of the ionospheric variation can be ignored. However, the impact of ionospheric delay will be amplified 10–120 times in the combination of phases and pseudoranges. If the variation of ionospheric delay is not corrected, it will cause a significant impact on the combination of phases and pseudoranges [37,38,39,40].

On the other hand, the characteristic of ionospheric variation can be investigated from the second-order difference of ionospheric delay:

(8) $$\Delta \Delta {\mathrm{iono}}_{1,k}^{}=\Delta {\mathrm{iono}}_{1,k}^{}-\Delta {\mathrm{iono}}_{1,k-1}^{}$$

For an observation interval of 30 s, mean of the second-order difference of ionospheric delay is about 0.000002 m. The ratio of the first-order difference to the second-order difference is about 2100. It shows that $\Delta \Delta {\mathrm{iono}}_{1,k}^{}\to 0$. Therefore, the ionospheric delay variation $\Delta {\mathrm{iono}}_{1,k}^{}$ at the current epoch can be corrected by the previous epoch $\Delta {\mathrm{iono}}_{1,k-1}^{}$, and the correction accuracy is demonstrated by $\Delta \Delta {\mathrm{iono}}_{1,k}^{}$.

In terms of ionospheric variation correction, the CSD condition is deduced. As a precondition, the CSD method with geometry-free phase assumes that the first-order difference of ionospheric delay approaches 0. In this study, a lower ionospheric effect on CSR methods is achieved since a stricter precondition is assumed: that the second-order difference of ionospheric delay approaches 0. Then, the ionospheric variation observed in the previous epoch is used to correct the ionospheric variation in the current epoch [23,30,34]. To start, a preprocessing is suggested. CSD with geometry-free phase is operated on the starting observation data to find the first epoch without cycle slip. For the phase data where the cycle slip occurred before the “first” epoch, it is still recommended to use the classic ambiguity re-initialization method to process the cycle slip. The first epoch without cycle slip is selected as the starting epoch of CSR, and the current ionospheric variation is calculated to correct the ionospheric variation in the next epoch. In turn, the ionospheric variation on the subsequent epoch will be corrected from the CSR results on the previous epoch. Through the correction of ionospheric variation, the significant influence of ionospheric delay on cycle slip calculation is weakened, especially when the ionospheric delay is amplified after the phases’ and pseudoranges’ combination, and its correction effect is also amplified simultaneously. Therefore, it is ensured that the amplified influence of the ionospheric variation is effectively corrected in the triple-frequency combination.

### 2.3. Optimization of Cycle Slip Solution from Minimizing the Total Noises after Ionospheric Correction

From Equation (3), the precision of the cycle slip solution is analyzed. A smaller variance in cycle slip solution can be resulted from the following factors: a longer wavelength, and a smaller influence of phase noise, pseudorange noise, and residual noise after the ionospheric correction, help to reach a higher precision of cycle slip solution. The above demands can be realized by the combination with triple-frequency observations. 

A longer wavelength can be obtained from a combined phase $\phi_{c}^{}$ with triple-frequency phases. A lower noise level can be achieved by a combined pseudorange $p_{c}^{}$ with triple-frequency pseudoranges. Then, the combined cycle slip $\Delta N_{c}^{}$ is built by the combination coefficients, which should result in a smaller variance $\sigma_{\Delta N_{c}^{}}^{2}$.

In order to get the combination coefficients, an optimal function with the objective of minimizing the $\sigma_{\Delta N_{c}^{}}^{2}$ is established as:

(9) $$\min(\frac{2\sigma_{p_{c}^{}}^{2}}{\lambda_{c}^{2}}+2\sigma_{\phi_{c}^{}}^{2}+\sigma_{c,\Delta iono}^{2})$$

and a geometry-free constraint is attached:

(10) $$\sum_{j=1}^{3}\frac{\lambda_{c}^{}}{\lambda_{j}^{}}m_{j}^{}-\sum_{j=1}^{3}n_{j}^{}=0$$

where $m_{j}^{}$ (*j* = 1,2,3) are the coefficients in the phase combination; $f_{j}^{}$ (*j* = 1,2,3) denote the basic frequencies on BDS carriers of B1, B2, B3 respectively; $\phi_{j}$ represents the phase on the frequency *j*; $\phi_{c}^{}=\sum_{j=1}^{3}m_{j}^{}\phi_{j}$ is the phase combination; *c* is the velocity of light; $\lambda_{c}^{}=c/(\sum_{j=1}^{3}m_{j}^{}f_{j}^{})$ represents the wavelength of the phase combination; $n_{j}^{}$ (*j* = 1,2,3) are the coefficients in the pseudorange combination; $p_{j}^{}$ represents the pseudorange on the frequency *j*; $p_{c}^{}=\sum_{j=1}^{3}n_{j}^{}p_{j}^{}$ is the pseudorange combination; $\Delta N_{c}^{}=\sum_{j=1}^{3}m_{j}^{}\Delta N_{j}$ is the combined cycle slip; $\sigma_{\Delta N_{c}^{}}^{2}$ is variance of $\Delta N_{c}^{}$; $\sigma_{\phi_{{}_{j}}^{}}^{}$ is the standard deviation of $\phi_{j}$; $\sigma_{\phi_{c}}^{2}=\sum_{j=1}^{3}\sigma_{\phi_{{}_{j}}^{}}^{2}m_{j}^{2}$ is the variance of $\phi_{c}^{}$, and the unit of $\sigma_{\phi_{c}}^{}$ is cycle; $\sigma_{p_{{}_{j}}^{}}^{}$ is the standard deviation of $p_{j}^{}$; $\sigma_{P_{c}^{}}^{2}=\sum_{j=1}^{3}\sigma_{p_{{}_{j}}^{}}^{2}n_{j}^{2}$ is the variance of $p_{c}^{}$, and the unit of $\sigma_{p_{c}}^{}$ is meter; $\sigma_{j,\Delta iono}^{}$ is the variance of ionospheric variation correction on the frequency *j* with the unit of meter; $\sigma_{c,\phi ,\Delta iono}^{2}=\sum_{j=1}^{3}(\sigma_{j,\Delta iono}^{}m_{j}^{}/\lambda_{j}^{}{)}_{}^{2}$ is the variance of ionospheric variation correction on the phase combination; $\sigma_{c,p,\Delta iono}^{2}=\sum_{j=1}^{3}(\sigma_{j,\Delta iono}^{}n_{j}^{}/\lambda_{c}^{}{)}_{}^{2}$ is the variance of ionospheric variation correction on the pseudorange combination; $\sigma_{c,\Delta iono}^{2}=\sigma_{c,\phi ,\Delta iono}^{2}+\sigma_{c,p,\Delta iono}^{2}$ is the variance of ionospheric variation correction on the cycle slip solution.

Obviously, Equation (9) is an objective function, and Equation (10) is a constraint function. In order to solve the Equation (9) under the condition of Equation (10), the traversal search method is suggested as a simple but effective way. The integer range in search of $m_{j}^{}$ (*j* = 1,2,3) is [−10,10], and the integer larger than 10 is discarded because of the magnified phase noise. The floating range in search of $n_{j}^{}$ (*j* = 1,2,3) is [−1,1] with the discretization step of 0.1, and the float larger than 1 is discarded because of the magnified pseudorange noise. For the purpose of keeping the combined ambiguities linearly independent, three combinations for cycle slip solution are searched. 

For conveniences of description and expression as listed in Table 1, the optimized phase combinations are noted as $\phi_{7,i}=\sum_{j=1}^{3}m_{i,j}^{}\phi_{j}$, and the corresponding ambiguity is noted as $N_{7,i}=\sum_{j=1}^{3}m_{i,j}^{}N_{j}$; $m_{i,j}^{}$ (*i* = 1,2,3; *j* = 1,2,3) are the coefficients of phase combinations; the wavelength of $\phi_{7,i}$ is noted as $\lambda_{7,i}^{}=c/(\sum_{j=1}^{3}m_{i,j}^{}f_{j}^{})$; the standard deviation of observation noise in $\phi_{7,i}$ is noted as $\sigma_{\phi_{7,i}}^{}=\sqrt{\sum_{j=1}^{3}\sigma_{\phi_{j}}^{2}m_{i,j}^{2}}$; the ionospheric scale factor in carrier $\phi_{7,i}$ can be noted as $ISFC_{7,i}=\sum_{j=1}^{3}\frac{f_{1}^{2}}{f_{j}^{2}\lambda_{j}^{}}m_{i,j}^{}$ (with respect to (wrt): B1, cycle), so the ionospheric delay in $\phi_{7,i}$ is ${\mathrm{iono}}_{1}^{}\times ISFC_{7,i}$; the standard deviation of the ionospheric correction in $\phi_{7,i}$ is noted as $\sigma_{7,i,\phi ,iono}^{}=\sqrt{\sum_{j=1}^{3}(\sigma_{1,iono}^{}\frac{f_{1}^{2}m_{i,j}^{}}{\lambda_{j}^{}f_{j}^{2}}{)}_{}^{2}}$. 

Accordingly, three pseudorange combinations corresponded to $\phi_{7,i}$ (*i* = 1,2,3) are noted as $p_{7,i}$ (*i* = 1,2,3) listed in Table 2, where $p_{7,i}=\sum_{j=1}^{3}n_{i,j}^{}p_{j}^{}$; $n_{i,j}^{}$ (*i* = 1,2,3; *j* = 1,2,3) are the coefficients of pseudorange combinations; the standard deviation of observation noise in $p_{7,i}$ is noted as $\sigma_{p_{7,i}}^{}=\sqrt{\sum_{j=1}^{3}\sigma_{p_{j}}^{2}n_{i,j}^{2}}$; the ionospheric scale factor in pseudorange $p_{7,i}$ can be noted as $ISFP_{7,i}=\sum_{j=1}^{3}\frac{f_{1}^{2}}{f_{j}^{2}}n_{i,j}^{}$ (wrt: B1, m), so the ionospheric delay in $p_{7,i}$ is ${\mathrm{iono}}_{1}^{}\times ISFP_{7,i}$; and the standard deviation of the ionospheric correction in $p_{7,i}$ is noted as $\sigma_{7,i,p,iono}^{}=\sqrt{\sum_{j=1}^{3}(\sigma_{1,iono}^{}\frac{f_{1}^{2}n_{i,j}^{}}{f_{j}^{2}}{)}_{}^{2}}$. The standard deviation of $N_{7,i}^{}$ without ionospheric correction is denoted as $\sigma_{N_{7,i}}^{}=\sqrt{\frac{\sigma_{p_{7,i}^{}}^{2}}{\lambda_{7,i}^{2}}+\sigma_{\phi_{7,i}}^{2}}$.

Finally, the ionospheric scale factor in $N_{7,i}$ is noted as $ISF_{7,i}=ISFC_{7,i}+ISFP_{7,i}/\lambda_{7,i}^{}$ (wrt: B1,cycle), so the ionospheric correction in $N_{7,i}$ is ${\mathrm{iono}}_{1}^{}\times ISF_{7,i}$. The standard deviation of the ionospheric correction in $\Delta N_{7,i}$ is noted as $\sigma_{7,i,c,\Delta \mathrm{iono}}^{}=\sqrt{\sigma_{7,i,p,\Delta iono}^{2}/\lambda_{7,i}^{2}+\sigma_{7,i,\phi ,\Delta iono}^{2}}$ (wrt: B1,cycle). The standard deviation of $\Delta N_{7,i}$ is denoted as $\sigma_{\Delta N_{7,i}}^{}=\sqrt{\frac{2\sigma_{p_{7,i}^{}}^{2}}{\lambda_{7,i}^{2}}+2\sigma_{\phi_{7,i}}^{2}+\sigma_{7,i,c,\Delta \mathrm{iono}}^{2}}$.

### 2.4. Cycle Slip Solution

Using the optimized phase combinations $\phi_{7,i}$ (*i* = 1,2,3) and corresponding pseudorange combinations $p_{7,i}$ (*i* = 1,2,3), the combined cycle slip is deduced as:

(11) $$\Delta N_{7,i}^{}=\frac{\Delta p_{7,i}^{}}{\lambda_{7,i}^{}}-\Delta \phi_{7,i}^{}-ISF_{7,i}\Delta {\mathrm{iono}}_{1,k-1}^{}$$

The float of $\Delta N_{7,i}$ and their covariance matrix can be calculated from (11). The integer least squares technique is used to solve Equation (11). LAMBDA, with high efficiency and excellent performance [16], is utilized to search the integer of $\Delta N_{7,i}$, employing the float estimates of $\Delta N_{7,i}$ and its covariance matrix as input. After the integer of $\Delta N_{7,i}$ is fixed, cycle slips on basic carriers $\Delta N_{j}$ (*j* = 1,2,3) can be easily calculated by:

(12) $$\left[\begin{array}{c} \Delta N_{1}\\ \Delta N_{2}\\ \Delta N_{3} \end{array}\right]={\left[\begin{array}{ccc} 0 & -1 & 1\\ 3 & -5 & 1\\ 4 & -1 & -4 \end{array}\right]}_{}^{-1}\left[\begin{array}{c} \Delta N_{7,1}\\ \Delta N_{7,2}\\ \Delta N_{7,3} \end{array}\right]={\left[\begin{array}{ccc} 21 & \;-5 & 4\\ 16 & -4 & 3\\ 17 & -4 & 3 \end{array}\right]}_{}^{}\left[\begin{array}{c} \Delta N_{7,1}\\ \Delta N_{7,2}\\ \Delta N_{7,3} \end{array}\right]$$

It shows that the combination matrix consisted of combination coefficients $m_{i,j}^{}$ (*i* = 1,2,3; *j*= 1,2,3), and possesses a good feature that elements of the inverse of the combination matrix are integers too.

### 2.5. Cycle Slip Repair Against Pseudorange Error

In the BDS observations, the precision of pseudorange is low because of a high level of observation noise, and large error may exist in the pseudorange. Analyzing Equation (11), the floating value of the combined cycle slip is calculated from the pseudoranges and phases. It can be seen that pseudorange error will affect the calculation of the combined cycle slip. If gross error occurs in pseudorange, there may be significant deviation in the calculation of the combined cycle slip. 

When the floats and covariance matrix are input, LAMBDA will output the integers with the smallest distance from the floats under the statistical space represented by the covariance matrix, and several candidate integers are supplied by the distance sorting. Thus, no significant deviation existing in the input floats is the premise of correctly using LAMBDA to the caller. If there is a large deviation in the float of the combined cycle slip, the initial reference to search the nearest integer has deviated. At this time, the actual true value may be located in the candidate values provided by LAMBDA. In proceeding, it is necessary to select the correct integer of the combined cycle slip among the candidate values by eliminating the interference of the pseudorange error.

Only using the original carrier phases, an ionosphere-free and geometry-free phase can be designed to offset geometric distance and ionospheric delay [41], and it is denoted as:

(13) $$L_{8}=\frac{f_{1}^{2}(\phi_{1}+N_{1})\lambda_{1}-f_{2}^{2}(\phi_{2}+N_{2})\lambda_{2}}{f_{1}^{2}-f_{2}^{2}}-\frac{f_{1}^{2}(\phi_{1}+N_{1})\lambda_{1}-f_{3}^{2}(\phi_{3}+N_{3})\lambda_{3}}{f_{1}^{2}-f_{3}^{2}}$$

An inherent constraint can be found in the ionosphere-free and geometry-free phase [41]. The ionosphere-free and geometry-free characteristic shows that the geometric distance, ionospheric delay, clock error, orbit error, tropospheric delay, earth rotation effect and relativity effect are cancelled. Since slowly changed instrumental bias and multipath error can be alleviated by time difference between adjacent epochs, the epoch-difference $L_{8}$ without cycle slip should come close to 0:

(14) $$\Delta L_{8}^{}\to 0$$

$\Delta L_{8}^{}$ can be obtained from original phase $\Delta \phi_{j}$ and $\Delta N_{j}^{}$, where $\Delta N_{j}^{}$ is calculated by Equations (11) and (12). The LAMBDA method can search several integer sets of $\Delta N_{7,i}(n)$ that can be converted to corresponding basic cycle slip $\Delta N_{j}^{}(n)$, where *n* is the index number of the candidate cycle slip combination. $\Delta L_{8}^{}(n)$ is calculated from the corresponding $\Delta N_{j}^{}(n)$. Since the time difference of the ionosphere-free and geometry-free phase approaches 0, the selection for correct cycle slip can follow this discrimination function:

(15) $$\min(\left|\Delta L_{8}^{}(n)\right|)$$

where the cycle slip value resulting in the minimum of absolute $\Delta L_{8}^{}$ is proposed as the final cycle slip. From the discrimination function, the minimum value is sorted by the absolute epoch-difference values of the ionosphere-free and geometry-free phases calculated by the cycle slip candidates, then the corresponding values of cycle slip are selected as the final values.

According to Equation (1), in order to keep the ambiguity unchanged, the phase value at the current epoch only needs to subtract the correction value of cycle slip and complete CSR, where the correction value of cycle slip should be the sum of the current cycle slip value and all previous cycle slip values. By subtracting the correction value of cycle slip from the current phase, the repair is accomplished.

### 2.6. Repair Result Checking

Whether the phase data can be directly used depends on the correctness of CSR results. Therefore, it is necessary to check the correctness of CSR results. The ionosphere-free and geometry-free phase can be used to check CSR results according to its epoch-difference value and variance. From the CSR results, the epoch-difference of ionosphere-free and geometry-free phase is calculated as:

(16) $$\Delta L_{8}=\frac{f_{1}^{2}(\Delta \phi_{1}+\Delta N_{1})\lambda_{1}-f_{2}^{2}(\Delta \phi_{2}+\Delta N_{2})\lambda_{2}}{f_{1}^{2}-f_{2}^{2}}-\frac{f_{1}^{2}(\Delta \phi_{1}+\Delta N_{1})\lambda_{1}-f_{3}^{2}(\Delta \phi_{3}+\Delta N_{3})\lambda_{3}}{f_{1}^{2}-f_{3}^{2}}$$

If $\left|\Delta L_{8}\right|<3\sqrt{2}\delta_{L_{8}}^{}$, then cycle slip is repaired successfully. Otherwise, the repair fails and the repair results are not available.

## 3. Test Description

The following comparative schemes are applied in the testing. Scheme 1: BDS triple-frequency CSR method (BTCSR) is utilized to get the cycle slip values. BTCSR conducts the ionospheric correction on the $\Delta N_{7,i}$ (*i* = 1,2,3), and the discrimination function (15) is employed to determine the cycle slip. The CSR results after the selection are obtained. Scheme 2: The conventional triple-frequency CSR method (CTCSR) is utilized to get the cycle slip values. CTCSR conducts the ionospheric correction on the $\Delta N_{7,i}$ (*i* = 1,2,3), and the fixing results of $\Delta N_{7,i}$ (*i* = 1,2,3) with LAMBDA are directly transformed to the basic cycle slip and adopted as the final values. Thus the CSR results before the selection are provided from the CTCSR. Scheme 3: CSR without ionospheric correction on $\Delta N_{7,i}$ (*i* = 1,2,3) (WICSR) is utilized to get the cycle slip. Scheme 4: A conventional CSD method, with BDS triple-frequency geometry-free phases of B1-B3 and B2-B3 carriers (GFCSD), is employed to detect the cycle slip. Scheme 5: A conventional CSD method, with BDS triple-frequency Melbourne–Wubeena combinations (MWCSD), is employed to detect the cycle slip.

The CSR experiment is conducted with the triple-frequency BDS data. In this experiment, the BDS observation data is collected from the CUT0 station at the south latitude 32 degrees in the network of the Multi-GNSS Experiment (MGEX). In fact, the observation interval is the difference between the times of adjacent observation epochs. The time of observation epoch is an integer multiple of the observation interval. The observation interval is specified as 30 s over the period of 3 h, starting from UTC 2016/05/08 00:00:00. Because this period appeared in a higher geomagnetic index of 6 for the day, a moderate magnetic storm occurred with level of 6 or more, and the ionosphere was severely active. The cutoff elevation is set as 10 degrees. 

Small and special cycle slips are inserted in the phase data to validate the performances of CSR and CSD. Small slips of (1,0,0) and (1,1,0) cycles on (B1,B2,B3), which are difficult to be detected and processed, have been used by many researchers to check the performance of CSR methods. Testing of small cycle slips repair can validate whether BTCSR can correctly repair cycle slips that are difficult to be repaired. The special slips of (1,1,1) cycles on (B1,B2,B3) cannot be detected by the MW combination. The special slips of (0,59,62) cycles on (B1,B2,B3) are difficult to be detected correctly by the geometry-free phase. Testing of the special cycle slips repair can assess whether BTCSR can overcome the shortages of CSD, with the MW combination and the geometry-free phase, respectively. The CSR process involves observation data of the previous epoch and the current epoch. The phase data at the previous epoch remains unchanged. The known integers are added to the phase data at the current epoch as the cycle slip values. According to the known integer values, the cycle slip repair values output by the CSR method are shown, which demonstrate the CSR performance. In the same way, epoch-by-epoch cycle slip adding and repairing are tested on the entire observation data.

CSR and CSD experiments included all observable geosynchronous earth orbit (GEO), inclined geosynchronous orbit (IGSO), and medium earth orbit (MEO) satellites. In view of the paper length, we only list the comparison results of MEO satellite 11. Because the MEO has fast orbital motion, the elevation and ionospheric delays vary significantly at the same observation interval, and its CSR processing becomes more complicated and representative. The other results of GEO, IGSO, and MEO satellites can be seen in the Supplementary Materials.

## 4. Experimental Results and Analysis

The BTCSR is evaluated in this section. With observation data from the BDS tracking station, BTCSR is tested against the ionospheric disturbance during the time of high ionospheric activity, where a moderate magnetic storm of level 6 or more occurred. Besides this, the performance of BTCSR in the situation of the pseudorange with additional gross error is inspected. Finally, results of BTCSR are compared with the CTCSR, WICSR, MWCSD and GFCSD. 

### 4.1. Results of BTCSR Against the Small and Special Cycle Slip

BTCSR is tested by inserting cycle slips on the observation data without cycle slip. The special slips of (1,0,0), (1,1,0), (1,1,1), and (0,59,62) cycles on (B1,B2,B3) were inserted on the consecutive data in epoch-by-epoch mode. BTCSR, GFCSD, and MWCSD are executed to detect or repair the inserted cycle slips.

**Figure 2:**
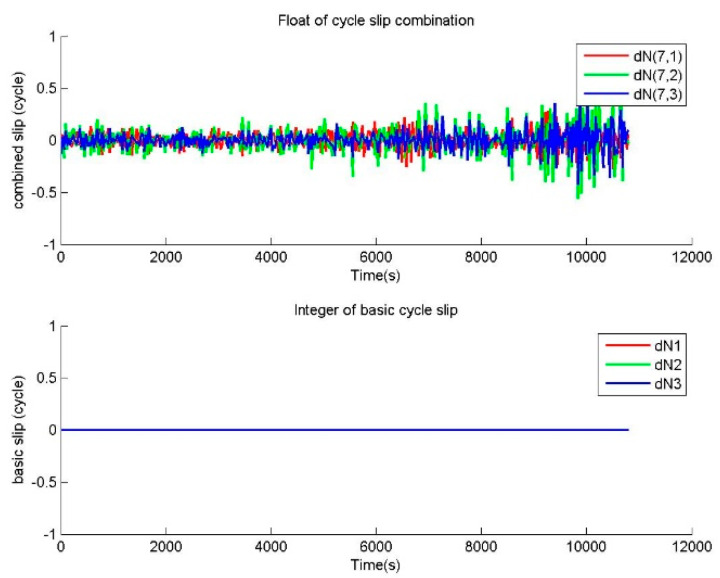
Values of cycle slip combinations and cycle slips on the basic frequencies provided by BTCSR in the case of the phase data without cycle slips, where the observation data was collected by CUT0 at south latitude of 32 degrees started from UTC 2016/05/08 00:00:00, BTCSR is the BDS triple-frequency cycle slip repair method.

**Figure 3:**
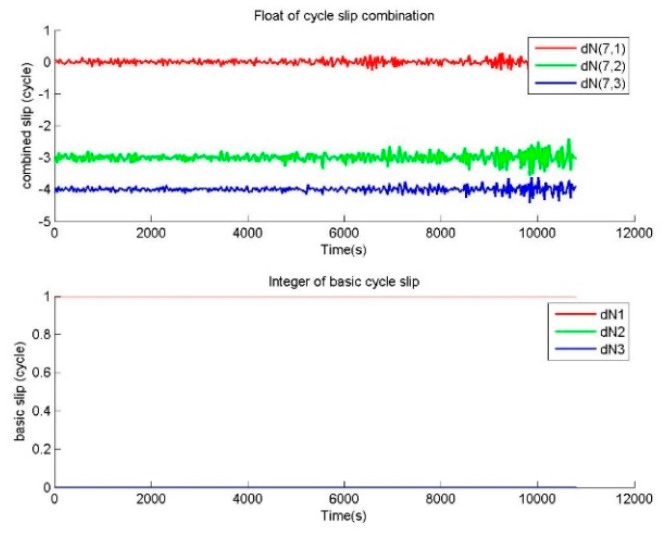
Values of cycle slip combinations and cycle slips on the basic frequencies provided by BTCSR in the case of the phase data with the known slips of (1,0,0) cycles on (B1,B2,B3), where the observation data was collected by CUT0 at south latitude of 32 degrees started from UTC 2016/05/08 00:00:00.

**Figure 4:**
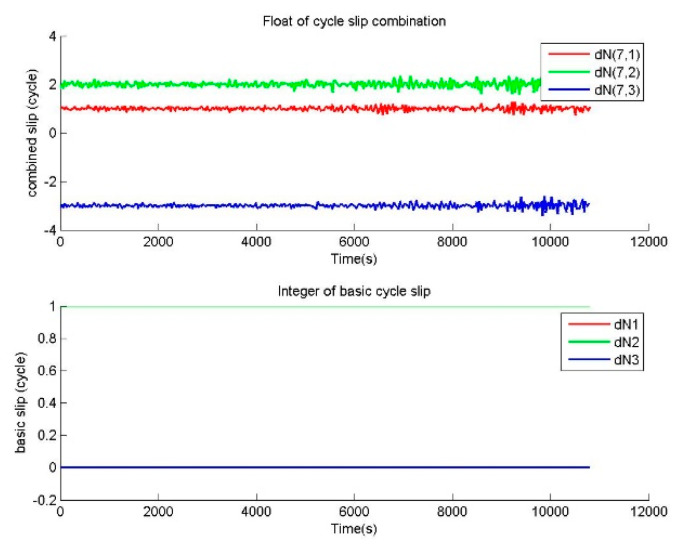
Values of cycle slip combinations and cycle slips on the basic frequencies provided by BTCSR in the case of the phase data with the known slips of (1,1,0) cycles on (B1,B2,B3), where the observation data was collected by CUT0 at south latitude of 32 degrees started from UTC 2016/05/08 00:00:00.

One repair testing is performed to verify the correctness of BTCSR in the repair of small cycle slips. For the observation data without cycle slip, small slips of (1,0,0), (1,1,0) cycles were added in the phase data. The cycle slip values determined by BTCSR are drawn as sequence diagrams. In the cases of no slip and slips of (1,0,0), (1,1,0) cycles, the floats of cycle slip combinations and the integers of basic cycle slips from BTSCR are shown in Figure 2, Figure 3 and Figure 4.

**Figure 5:**
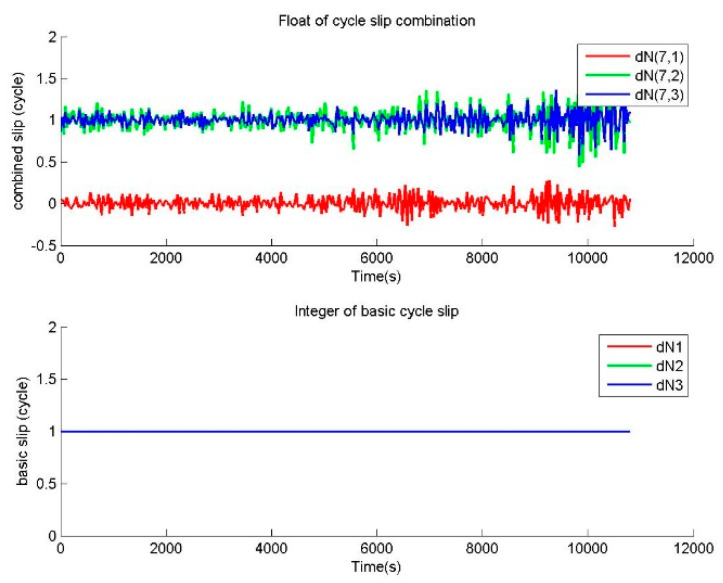
Values of cycle slip combinations and cycle slips on the basic frequencies provided by BTCSR in the case of the phase data with the known slips of (1,1,1) cycles on (B1,B2,B3), where the observation data was collected by CUT0 at south latitude of 32 degrees started from UTC 2016/05/08 00:00:00.

**Figure 6:**
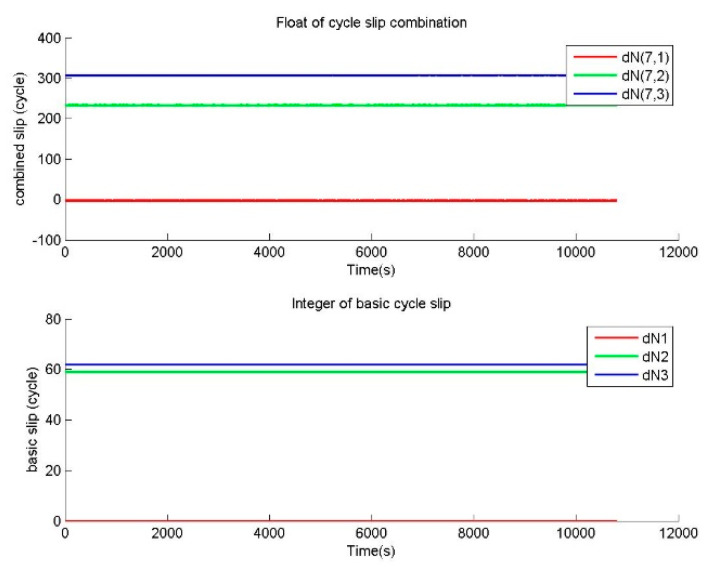
Values of cycle slip combinations and cycle slips on the basic frequencies provided by BTCSR in the case of the phase data with the slips of (0,59,62) cycles on (B1,B2,B3), where the observation data was collected by CUT0 at south latitude of 32 degrees started from UTC 2016/05/08 00:00:00.

The other repair testing is performed to verify the correctness of BTCSR in the repair of special cycle slips. For the observation data without cycle slip, special slips of (1,1,1) and (0,59,62) cycles on (B1,B2,B3) were added in the phase data. The cycle slip values determined by BTCSR are drawn as sequence diagrams. In the cases of cycle slips (1,1,1) and (0,59,62), the floats of cycle slip combinations and the integers of basic cycle slips from BTSCR are shown in Figure 5 and Figure 6. 

The observation data without cycle slip is used to calculate the value of cycle slip combination, and Figure 2 shows that absolute values of cycle slip combinations calculated by BTCSR are basically within 0.5. It means that, after the correcting of ionospheric variation, the combinations of phases and pseudoranges obtained by minimizing total noises are capable of calculating the cycle slip combinations. Figure 2 also shows that the integer values of basic cycle slips supplied by BTCSR are all 0. These results are consistent with the actual situation of the observation data without cycle slips. It indicates that BTSCR can correctly fix the cycle slip value. 

The observation data with cycle slips of (1,0,0), (1,1,0), (1,1,1) cycles on (B1,B2,B3) is used to calculate the value of cycle slip combination, and Figure 3, Figure 4 and Figure 5 show that the absolute values of cycle slip combinations calculated by BTCSR are significantly changed to 2–4. It indicates that the combinations of phases and pseudoranges are more sensitive to small cycle slips, and BTSCR has a better identification performance for small cycle slips. The integer values of basic cycle slips provided by BTSCR show that all additional small cycle slips are correctly fixed. As such, the performance of BTSCR is correct and reliable for repairing small cycle slips.

In the case of the special cycle slips (N,N,N), where N is the integer, the correctness of BTCSR can be compared with MWCSD. The observation data with cycle slips (1,1,1) is used to calculate the cycle slip combinations of BTCSR and MWCSD. MWCSD fails to detect the cycle slips (1,1,1). Because equal cycle slips on basic frequencies are indicated as 0 in the single-difference phase between basic frequencies, (N,N,N) is undetectable by the CSD and CSR with the single-difference phase between frequencies. However, Figure 5 shows that the BTCSR results are still consistent with the known values of cycle slips.

In the case of the special cycle slips (0,59N,62N), the correctness of BTCSR can be compared with GFCSD. The observation data with cycle slips of (0,59,62) is used to calculate the cycle slip combinations by BTCSR, and the geometry-free phases by GFCSD. GFCSD fails to correctly detect cycle slips of (0,59,62). Since the fact is $59N\lambda_{2}^{}=62N\lambda_{3}^{}$, and the product that these cycle slips multiply by the corresponding basic wavelengths are the same on the carriers. In this situation, the cycle slips (0,59N,62N) are indicated as 0 in the B2–B3 geometry-free phase. So GFCSD is invalid for these cycle slips. However, Figure 6 shows that the BTCSR results are still consistent with the known values of cycle slips. 

### 4.2. Results of BTCSR Against the Severe Ionospheric Variation

**Figure 7:**
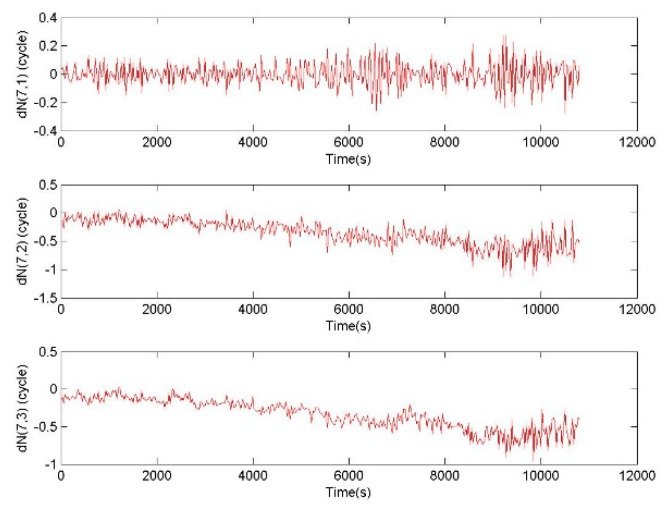
Values of cycle slip combinations provided by WICSR, in the case of the phase data without cycle slip and the ionospheric variation without correcting, where the observation data was collected by CUT0 at south latitude of 32 degrees started from UTC 2016/05/08 00:00:00, WICSR is the cycle slip repair method without ionospheric correction.

During a moderate magnetic storm with a level of 6 or above, BTCSR and WICSR are tested, where WICSR ignores the ionospheric variation. For a BDS MEO observation data without cycle slip, WICSR gets the sequence of cycle slip combinations shown in Figure 7. The $\left|\Delta N_{7,i}\right|$ (*i* = 1,2,3) less than 0.5 can be fixed to 0, which is the true cycle slip integer. The $\left|\Delta N_{7,i}\right|$ (*i* = 1,2,3) greater than 0.5 will lead to repair failure because of fixing to an incorrect cycle slip integer. Figure 7 shows that the absolute values of $\Delta N_{7,2}$ and $\Delta N_{7,3}$ in WICSR gradually exceed 0.5 to 1. It means that WICSR exhibits a failure, because the cycle slip combination is significantly affected by the ionospheric delay variation.

**Figure 8:**
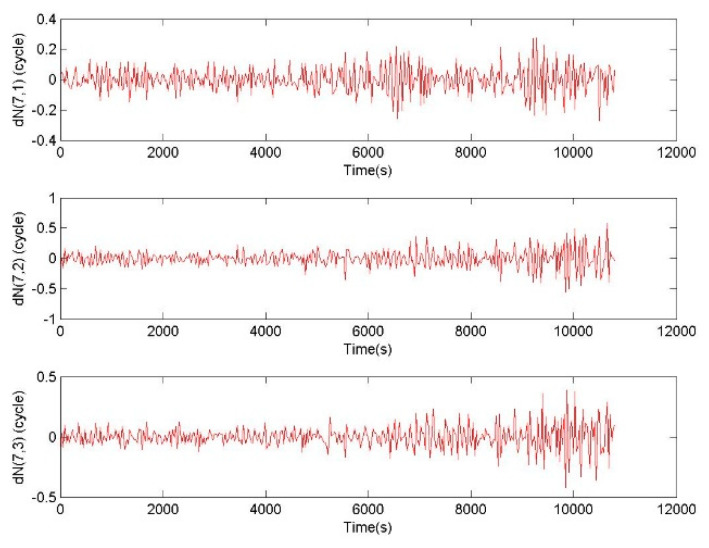
Values of the cycle slip combinations provided by BTCSR after correcting ionospheric variation from the phase data without cycle slip, where the observation data was collected by CUT0 at south latitude of 32 degrees started from UTC 2016/05/08 00:00:00.

For the same observation data without cycle slip, BTCSR gets the sequence of cycle slip combinations shown in Figure 8. After correcting the effect of the ionospheric delay variation, the average values of $\Delta N_{7,2}$ and $\Delta N_{7,3}$ are close to 0, and the absolute values are almost within 0.2 to 0.5. It is shown that the ionospheric delay contained in the value of cycle slip combination is greatly weakened. The correction of ionospheric variation is effective in BTCSR. So BTCSR can be used for calculating the cycle slip combination.

Regarding the ability of resisting the ionospheric disturbance, the better experimental performance of BTCSR than WICSR may be caused by two reasons. The first reason is that BTCSR has corrected the ionospheric variation in $\Delta N_{7,i}$ (*i* = 1,2,3), and the effect of ionospheric correction residual is minimized by the optimization on the combination. The first-order ionospheric delay is corrected. The second-order ionospheric delay is treated as correction residual, and its influence on cycle slip is constrained by the minimization of cycle slip variance. The second reason is the appending of $\Delta L_{8}$ in BTCSR. Because the ionosphere-free feature is possessed in the $\Delta L_{8}$, the influence of ionospheric delay on the cycle slip determination is eliminated. If $\Delta N_{j}$ is contaminated by the ionospheric variation residual, $\left|\Delta L_{8}\right|$ calculated from $\Delta N_{j}$ will significantly deviate from 0 and increase, and the larger $\left|\Delta L_{8}\right|$ is discarded because of the sorting rule. 

### 4.3. Results of BTCSR Against the Large Pseudorange Error

Similar to the MWCSD and CTCSR, the performance of BTCSR is influenced by the noise of pseudorange, and it is necessary to test the ability of the CSR method in resisting the pseudorange error. Since the original pseudorange has contained the random noise or even the multipath error, analysis of the additional errors on the real data is a supplementary way to test the upper limit of BTCSR on resisting the gross errors on the actual pseudoranges. Then, the CSR testing is performed under the normal real pseudorange and the epoch-difference pseudorange with additional error of 1.5 m, and the discrimination function of BTCSR is checked in light of eliminating the interference of pseudorange error and ensuring the reliability of CSR.

In the case of the observation data without cycle slip from CUT0 station, the additional errors of (1.5,1.5,1.5) m were imposed on all original epoch-difference triple-frequency pseudoranges. Considering the root mean squared error (RMS) of the pseudorange is 0.3 m, the RMS of the epoch-difference pseudorange is 0.424 m. The random errors of 99.96% are within three times RMS, and the three times RMS of the epoch-difference pseudorange is 1.273 m. Therefore, 1.5 m can almost cover the epoch-difference pseudorange errors of 99.96%. Before the selection with the discrimination function (15), CTCSR can directly output the initial cycle slip value $\Delta N_{j}$ from Equation (12), and the $\left|\Delta L_{8}\right|$ is calculated by $\Delta N_{j}$ from CTCSR results. After the selection with the discrimination function (15), BTCSR determines the final cycle slip value $\Delta N_{j}$ and $\left|\Delta L_{8}\right|$. The ratio is gotten from $\left|\Delta L_{8}\right|$ before selection dividing that of after selection, where a ratio of larger than 3 means the initial outputs from CTCSR are significantly disturbed by pseudorange error, and a ratio of 1 means the initial output is not disturbed by pseudorange error. 

**Figure 9:**
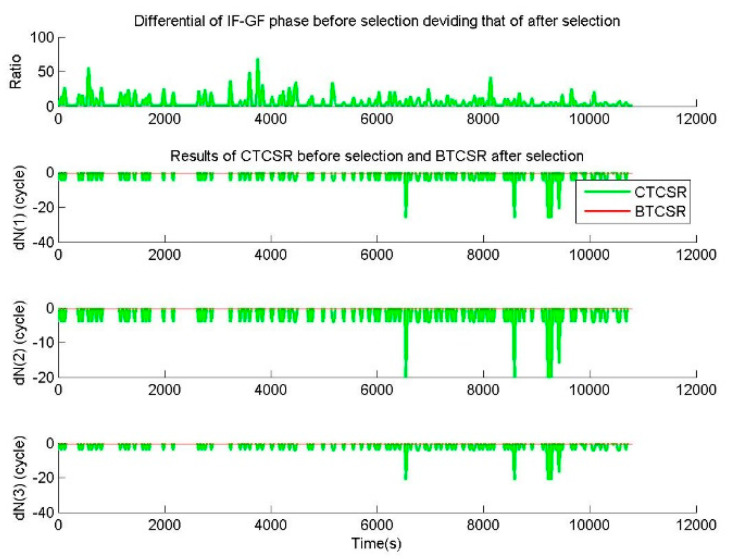
Values of cycle slips on basic frequencies provided by CTCSR before selection and BTCSR after selection in the case of the epoch-difference pseudoranges, with an additional error of 1.5 m, and the phases without cycle slip, where the observation data was collected by CUT0 at south latitude of 32 degrees started from UTC 2016/05/08 00:00:00, CTCSR is the conventional triple-frequency cycle slip repair (CSR) method, BTCSR is the BDS triple-frequency CSR method.

**Figure 10:**
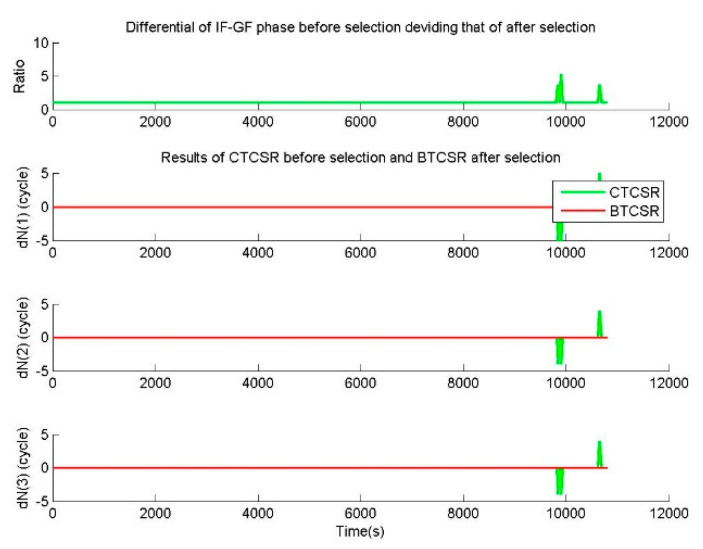
Values of cycle slips on basic frequencies provided by CTCSR before selection and BTCSR after selection, in the case of the normal real pseudoranges and the phases without cycle slip, where the observation data was collected by CUT0 at south latitude of 32 degrees started from UTC 2016/05/08 00:00:00.

For the phase data without cycle slip and the pseudorange data with additional 1.5 m errors, results of BTCSR and CTCSR are shown in Figure 9. It can be seen that due to the additional 1.5 m errors on the epoch-difference pseudoranges, the results of CTCSR display many failures, but the BTCSR results are correct and reliable. For normal pseudorange data and phase data without cycle slip, the results of BTCSR and CTCSR are shown in Figure 10. It can be seen that when the normal pseudoranges meet large errors because of the lower elevation, the CTCSR results encounter several failures, but the BTCSR results keep correct and reliable. 

The CTCSR results before the selection show that the cycle slips exhibit misjudgments, since the cycle slip is falsely fixed due to the interference of the pseudorange error. Further analyzing the sequence of floating values of the cycle slip combinations, some absolute values are between 0.5 and 0.7. It shows that noticeable deviations in the calculation of cycle slip combinations are caused by the pseudorange error. When the deviations from the cycle slip combinations exceed 0.5, the fixed result is incorrect. At this time, directly applying the fixed value of the cycle slip may cause a repair error.

The BTCSR results after the selection show that the cycle slips are determined correctly. Since the discrimination function has eliminated the disturbance of the pseudorange error, the reliable and correct repair results are achieved. Although some absolute values of the cycle slip combinations are between 0.5 and 0.7, the BTCSR results are not disturbed by the pseudorange errors. When the deviation from the absolute value exceeds 0.5, the discrimination function can select the correct cycle slip from the candidates.

**Figure 11:**
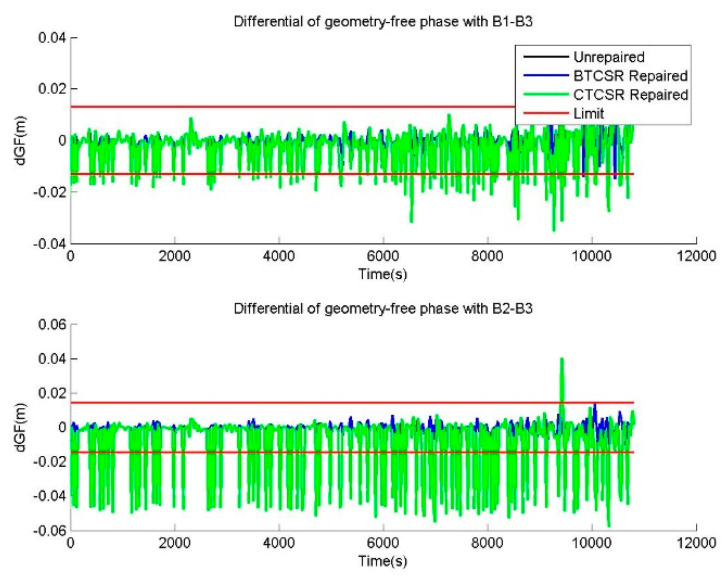
The differentials of geometry-free phases, provided by the unrepaired data and the repaired data with BTCSR and CTCSR in the case of epoch-difference pseudoranges, with additional 1.5 m errors and phases without cycle slip, where the observation data was collected by CUT0 at south latitude of 32 degrees started from UTC 2016/05/08 00:00:00.

**Figure 12:**
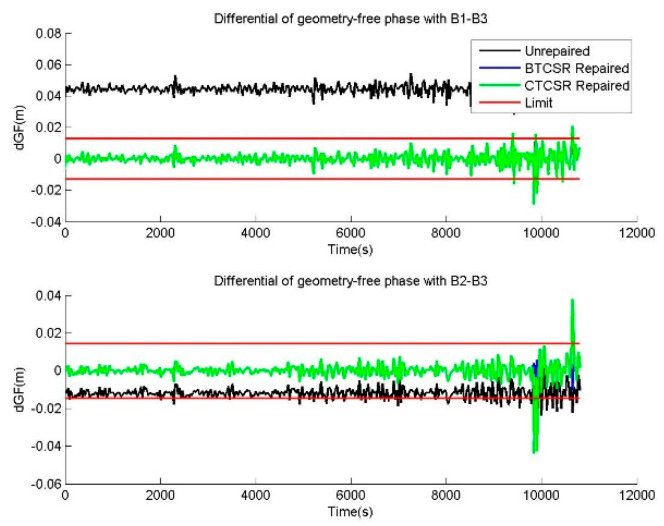
The differentials of geometry-free phases, provided by the unrepaired data and the repaired data with BTCSR and CTCSR in the case of normal pseudorange data and phase data with slips of (1,1,1) cycles on (B1,B2,B3), where the observation data was collected by CUT0 at south latitude of 32 degrees started from UTC 2016/05/08 00:00:00.

The raw phase data and the corrected phase data after the repair with BTCSR and CTCSR are compared and shown. The epoch-difference value of the geometry-free phase is usually employed to check the CSR effect, which demonstrates the influence of cycle slips contained in the phase data. As such, differentials of geometry-free phases from the raw phases, the phases after cycle slip corrections with BTCSR or CTCSR, can be drawn to compare the CSR performance. In the case of epoch-difference pseudoranges with additional 1.5 m errors and phases without cycle slip, the comparisons before and after cycle slip corrections with BTCSR and CTCSR are shown in Figure 11. In the case of normal pseudorange data and phase data with slips of (1,1,1) cycles on (B1,B2,B3), the comparisons before and after cycle slip corrections with BTCSR and CTCSR are shown in Figure 12. For other cycle slip corrections, the results are similar. From the results before and after cycle slip corrections with BTCSR and CTCSR, it can be seen that the performance of BTCSR is reliable, but that of CTCSR includes failures. The phase data after the cycle slip corrections by BTCSR is free from cycle slip. Since differentials of the geometry-free phase after the correction are, overall, within the limit, the BTCSR results are correct. On the other hand, many of the repaired results of CTCSR are correct, and overlapped with those of BTCSR. However, the phase data after the cycle slip corrections with CTCSR still contains several cycle slips. Because some differentials of the geometry-free phases after the corrections exceed the limit, the CTCSR fails to repair all cycle slips correctly.

A comparative test between BTCSR and CTCSR was carried out. The compared results show that BTCSR can still correctly repair cycle slips in the case of large pseudorange errors, but CTCSR shows the improper fixing of combined cycle slip and the failure to repair basic cycle slip due to the influence of large pseudorange errors. The large pseudorange error brings a significant deviation into the cycle slip combination calculated from the combination of phases and pseudoranges. When the deviation of the combined cycle slip from the correct integer is larger than 0.5, it is easy to cause the combined cycle slip to be fixed as an improper value that is discrepant from the actual correct value. In order to deal with the influence of significant deviation resulting from the large pseudorange error on the cycle slip fixing, BTCSR sets up a discriminant function. The candidates of cycle slip values are sorted according to the absolute epoch-difference value of the ionosphere-free and geometry-free phase, where the cycle slip corresponding to the minimum absolute value is selected as the final repair value. Since the discriminant function has eliminated the interference of pseudorange error and ionospheric delay on determining the actual cycle slip, the CSR correctness is guaranteed. This shows the improvement of BTCSR.

**Figure 13:**
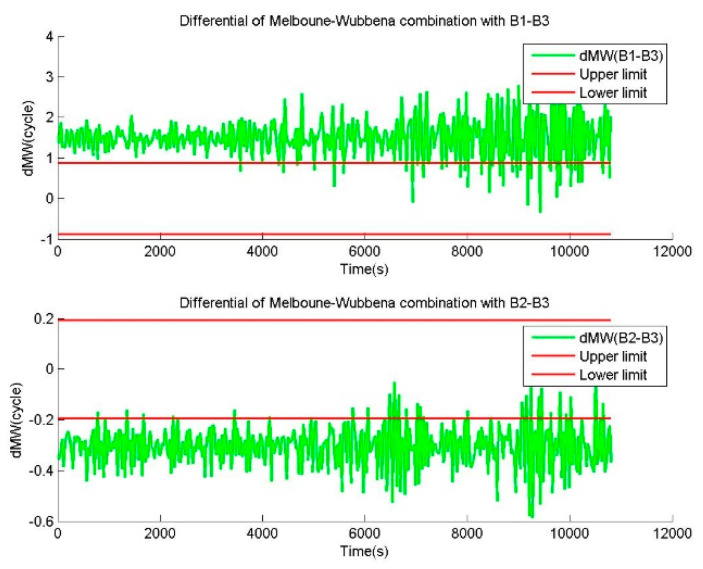
Values of cycle slip combinations provided by MWCSD in the case of the epoch-difference pseudoranges, with an additional error of 1.5 m and the phases without cycle slip, where the observation data was collected by CUT0 at south latitude of 32 degrees started from UTC 2016/05/08 00:00:00.

The combined cycle slips of MWCSD from the epoch-difference pseudoranges, with an additional error of 1.5 m, is shown in Figure 13. The results of MWCSD from the epoch-difference pseudoranges, with an additional error of 1.5 m and the phases without cycle slip, show that most detection results of MWCSD failed. Extensive cycle slip misjudgments occur in MWCSD, which indicates that the cycle slip combinations of MWCSD are significantly affected by pseudorange errors. Further analyzing the sequence of floating values of the cycle slip combinations calculated by MWCSD, most absolute values are between 1 and 3. All cycle slip combinations exceed the detection limits of MWCSD. It shows that noticeable deviations in the calculation of cycle slip combinations are caused by the pseudorange error. When the deviations exceed the detection limits, the detection results are incorrect.

For the ability of resisting the pseudorange error, the better experimental performance of BTCSR than CTCSR and MWCSD may be caused by two reasons. The first reason is the help of the selection function of $\min(\left|\Delta L_{8}\right|)$ in BTCSR. Because the pseudoranges are not involved in the $\Delta L_{8}$, the influence of pseudorange error on the BTCSR is eliminated by the selection function. The second reason is the long wavelengths and the low noise levels from the combinations in the BTCSR. On the one hand, the long wavelength can compress the pseudorange noise as a smaller phase noise in the carrier domain, thereby reducing the influence of the pseudorange error on the cycle slip calculation. On the other hand, the long wavelength can form a larger search step in the range domain; thereby the search space is expanded and the cycle slip determination is ensured correctly.

Theoretically, according to the RMS of (0.0646,0.1261,0.1069) cycles from the combined cycle slip calculation, the CSR success rate is 99.9927%, and the corresponding failure rate is 0.0073%. Fortunately, with the help of the discrimination function, for the observation data with elevation larger than 10 degrees, BTCSR can correctly repair all cycle slips, and the actual success rate is increased to 100%. However, for the severe observation environment with elevation of 5–10 degrees, the failure rate is about 0.037%, due to the occasional abnormal observation. Therefore, the observation environment with elevation larger than 10 degrees is recommended by BTCSR. From the analysis of the noise of pseudorange [35], the pseudorange noise is decreased with the satellite rise. As such, the cut-off elevation larger than 10 degrees is suggested to avoid the abnormal noise or gross error on the observation, and the lower elevation and bad observational environment, resulting in the larger noise, will challenge all CSD and CSR methods.

## 5. Conclusions

BDS has entered in third-generation times, and is providing a global service. More abundant resources of triple-frequency navigation signals can be obtained from BDS. For BDS phase data processing and application, cycle slip detection (CSD) is the primary work, since cycle slip repair (CSR) can reduce the cases of ambiguity initialization and the fluctuations of positioning results. There is much interest in exploiting the advantages of BDS triple-frequency resources to improve the CSR performance. By referencing the conventional triple-frequency CSR with the combinations of phases and pseudoranges (CTCSR) in correcting ionospheric delay and optimizing observational noises, a method of BDS triple-frequency CSR (BTCSR) was developed for the undifferenced phase. Compared to CTCSR, BTCSR brings the following improvements:

(1) On the basis of correcting the influence of the ionospheric variation, the effect of the residual ionospheric error after the correction of CSR is further minimized. Under the condition that the second-order difference of the ionospheric delay approaches 0, the first-order difference of the ionospheric delay at the previous epoch is used to correct the variation of ionospheric delay at the current epoch. After the ionospheric variation is corrected, the residual ionospheric influence is optimized. By minimizing the variance of the combined cycle slip, an optimal model of calculating cycle slip combination is formed, which also covers targets such as the smaller amplification effect of ionospheric correction error. Then, the calculation method is supplied for the combined cycle slip with the root mean squared errors of (0.0646,0.1261,0.1069) cycles, resulting in CSR success rate of 99.9927%, the ionospheric amplification coefficients with respect to cycle units of (0,12.0345,11.7112), and the wavelengths of (4.8842,3.5738,8.1403) m.
(2) The cycle slips provided by CTCSR have been further selected by BTCSR, and the CSR reliability is improved by eliminating the influence of larger pseudorange errors on determining the correct cycle slip. The large pseudorange error may lead to a significant deviation of the combined cycle slip calculated from the combination of phases and pseudoranges. When the deviation of the combined cycle slip from the correct integer is larger than 0.5, it easily causes the combined cycle slip to be fixed as an improper value that is discrepant from the actual correct value. In order to deal with the influence of significant deviation resulting from the large pseudorange error on the cycle slip fixing, BTCSR sets up a discriminant function. The candidates of cycle slip values are sorted according to the absolute epoch-difference value of the ionosphere-free and geometry-free phase, where the cycle slip corresponding to the minimum absolute value is selected as the final repair value. Since the discriminant function eliminates the interference of pseudorange error and ionospheric delay on determining the actual cycle slip, the CSR correctness is guaranteed, and the reliability is improved.

The compared testing shows that BTCSR achieves better performance than CTCSR in the situation of larger pseudorange errors. For the real BDS pseudorange observation with additional 1.5 m errors, which can cover the situations of 99.96% pseudorange noise, the results of CTCSR show failures, but the results of BTCSR keep correct. Conclusively, the reliability of BTCSR was verified from comparative testing and evaluation experiments, under conditions of moderate magnetic storm and additional pseudorange errors. Results of BTCSR with observation data in low latitudes show that BTCSR displays the characteristics of resisting the interferes of severe ionospheric variation and large pseudorange errors on CSR. Under the condition of appending 1.5 m gross error on the epoch-difference pseudoranges, the results of BTCSR are reliable, but the results of CTCSR and CSD with MW combination show failures. Meanwhile, under the environment of severe ionospheric variation during a moderate magnetic storm above level 6, the results of BTCSR are correct, but the results of CSD with geometry-free phase show failures. Furthermore, BTCSR overcomes the shortages of CSD with geometry-free phase or MW combination, which is invalid for special cycle slip, and can detect and repair the small slips (1,1,1) cycles or special slips (0,59,62) cycles on (B1,B2,B3). 

BTCSR has completed the evaluations in representative conditions, with the weather of moderate magnetic storm, the region of active ionosphere, and the observations of large pseudorange errors. However, it is difficult for one paper to traverse all extreme conditions and massive observation data. Processing with a large amount of observation data under static and kinetic environments can still be carried out to test the applicability of BTCSR in the future. The application of BTCSR has no special requirements for navigation ephemeris. Any restrictions on the number of observable satellites are not demanded either. BTCSR will be beneficial to CSR for fast and precise point positioning, precise navigation of BDS users, and so on.

## Figures and Tables

**Table 1:** The optimized phase combinations of triple-frequency BDS.

$\phi_{7,i}$	$m_{i,j}^{}$	$\lambda_{7,i}^{}\;(m)$	$\sigma_{\phi_{7,i}}^{}\;(\mathrm{cycles})$	$ISFC_{7,i}$ (wrt: B1,cycle)	$\sigma_{N_{7,i}}^{}\;(\mathrm{cycles})$
$\phi_{7,1}$	0, −1,1	4.8842	0.0141	−0.3258	0.0457
$\phi_{7,2}$	−3, 5, −1	3.5738	0.0592	11.6406	0.0768
$\phi_{7,3}$	−4, 1, 4	8.1403	0.0574	11.5382	0.0613

**Table 2:** The pseudorange combinations corresponding to the optimized phase combinations.

$p_{7,i}$	$n_{i,j}^{}$	$\sigma_{p_{7,i}}^{}\;(m)$	$ISFP_{7,i}$ (wrt: B1, m)	$ISF_{7,i}$ (wrt: B1, cycle)	$\sigma_{\Delta N_{7,i}}^{}\;(\mathrm{cycles})$
$p_{7,1}$	0,0.48760330578513, 0.51239669421487	0.2122	1.5915	0	0.0646
$p_{7,2}$	0.3, 0.3, 0.4	0.1749	1.4075	12.0345	0.1261
$p_{7,3}$	0.3, 0.3, 0.4	0.1749	1.4075	11.7112	0.1069

## Supplementary Materials

The following are available online at https://www.mdpi.com/1424-8220/20/10/2819/s1, Figures S1–S16: The Compared Results of Cycle Slip Repair from GEO/IGSO/MEO Satellites, Figures S17–S32: The Repaired Performances with Different Methods from GEO/IGSO/MEO Satellites.
