# Supplementary material for: A Cycle Slip Repair Method Against Ionospheric Effects and Observational Noises for BDS Triple-Frequency Undifferenced Phases
Source: Sensors (Basel). 2020 May 15;20(10):2819. doi: 10.3390/s20102819 (PMC7284426; doi:10.3390/s20102819)
Supplement: Supplementary file 1 [file sensors-20-02819-s001.pdf]

sensors

MDPI

1. The Compared Results of Cycle Slip Repair from GEO/IGSO/MEO Satellites

For the phase data without cycle slip and the epoch-difference pseudoranges with additional 1.5 m errors, results of BTCSR and CTCSR from GEO/IGSO/MEO satellites are shown in Figures S1–S12. For normal pseudorange data and phase data without cycle slip, results of BTCSR and CTCSR from IGSO/MEO satellites are shown in Figures S13–S16. Other CSR comparison results of special cycle slips of (1,0,0), (1,1,0), (1,1,1), (0,59,62) are similar.

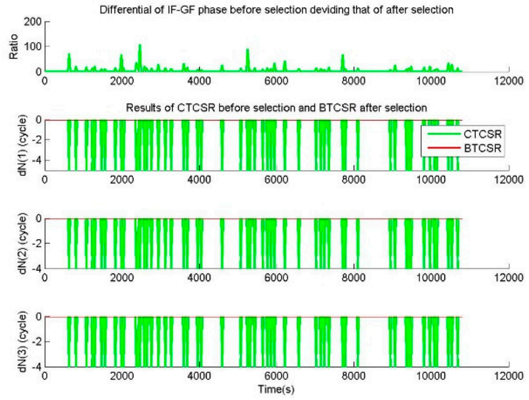

Figure S1. The cycle slip values provided from BTCSR and CTCSR to the GEO satellite 1 in the case of pseudorange data with additional errors of 1.5 m and phase data without cycle slip. Where the BDS data was collected by CUT0 at south latitude of 32 degrees, started from UTC 2016/05/08 00:00:00.

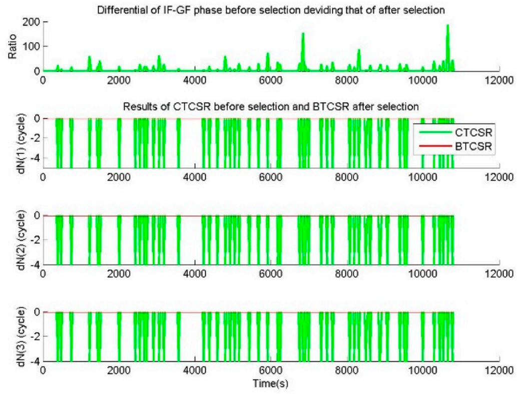

Figure S2. The cycle slip values provided from BTCSR and CTCSR to the GEO satellite 2 in the case of pseudorange data with additional errors of 1.5 m and phase data without cycle slip. Where the BDS data was collected by CUT0 at south latitude of 32 degrees, started from UTC 2016/05/08 00:00:00.

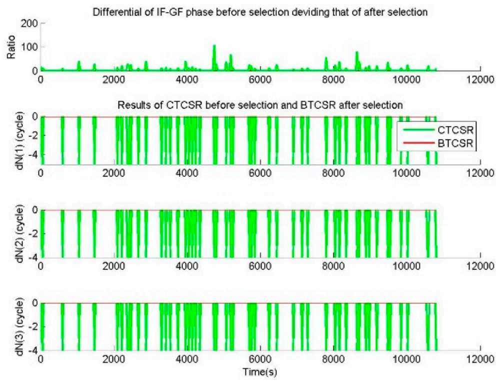

Figure S3. The cycle slip values provided from BTCSR and CTCSR to the GEO satellite 3 in the case of pseudorange data with additional errors of 1.5 m and phase data without cycle slip. Where the BDS data was collected by CUT0 at south latitude of 32 degrees, started from UTC 2016/05/08 00:00:00.

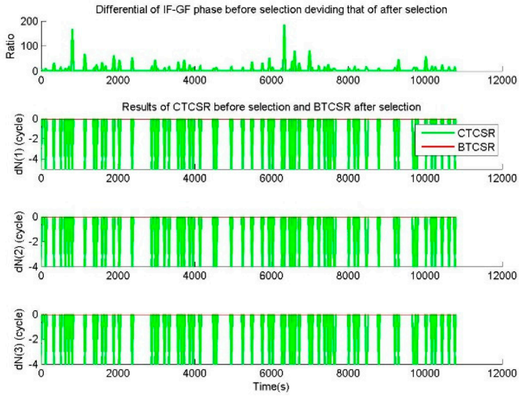

Figure S4. The cycle slip values provided from BTCSR and CTCSR to the GEO satellite 4 in the case of pseudorange data with additional errors of 1.5 m and phase data without cycle slip. Where the BDS data was collected by CUT0 at south latitude of 32 degrees, started from UTC 2016/05/08 00:00:00.

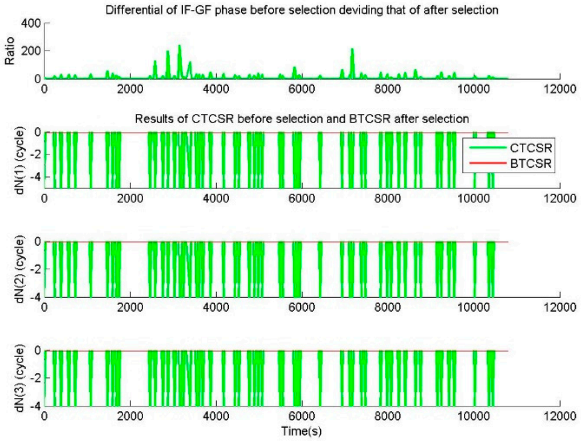

Figure S5. The cycle slip values provided from BTCSR and CTCSR to the GEO satellite 5 in the case of pseudorange data with additional errors of 1.5 m and phase data without cycle slip. Where the BDS data was collected by CUT0 at south latitude of 32 degrees, started from UTC 2016/05/08 00:00:00.

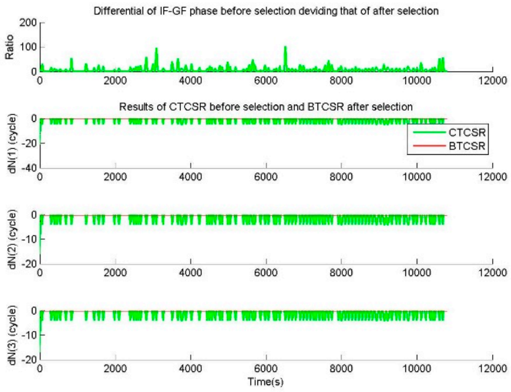

Figure S6. The cycle slip values provided from BTCSR and CTCSR to the IGSO satellite 6 in the case of pseudorange data with additional errors of 1.5 m and phase data without cycle slip. Where the BDS data was collected by CUT0 at south latitude of 32 degrees, started from UTC 2016/05/08 00:00:00.

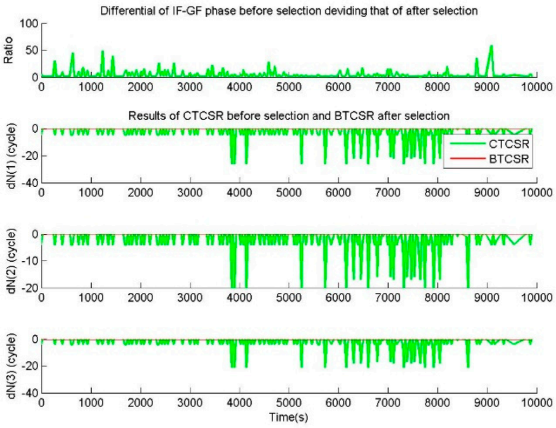

Figure S7. The cycle slip values provided from BTCSR and CTCSR to the IGSO satellite 7 in the case of pseudorange data with additional errors of 1.5 m and phase data without cycle slip. Where the BDS data was collected by CUT0 at south latitude of 32 degrees, started from UTC 2016/05/08 00:00:00.

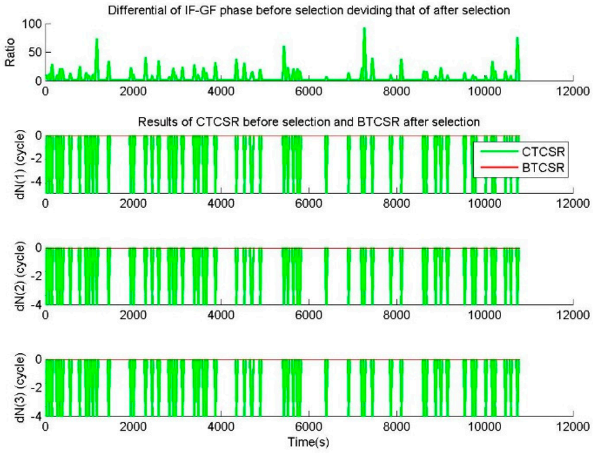

Figure S8. The cycle slip values provided from BTCSR and CTCSR to the IGSO satellite 8 in the case of pseudorange data with additional errors of 1.5 m and phase data without cycle slip. Where the BDS data was collected by CUT0 at south latitude of 32 degrees, started from UTC 2016/05/08 00:00:00.

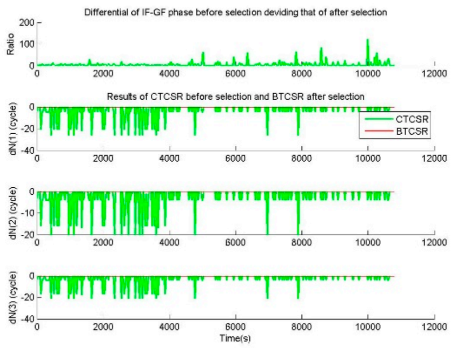

Figure S9. The cycle slip values provided from BTCSR and CTCSR to the IGSO satellite 9 in the case of pseudorange data with additional errors of 1.5 m and phase data without cycle slip. Where the BDS data was collected by CUT0 at south latitude of 32 degrees, started from UTC 2016/05/08 00:00:00.

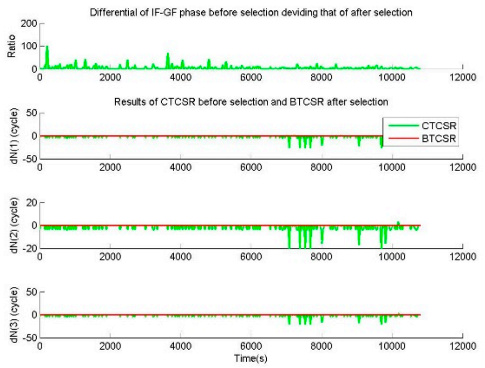

Figure S10. The cycle slip values provided from BTCSR and CTCSR to the IGSO satellite 10 in the case of pseudorange data with additional errors of 1.5 m and phase data without cycle slip. Where the BDS data was collected by CUT0 at south latitude of 32 degrees, started from UTC 2016/05/08 00:00:00.

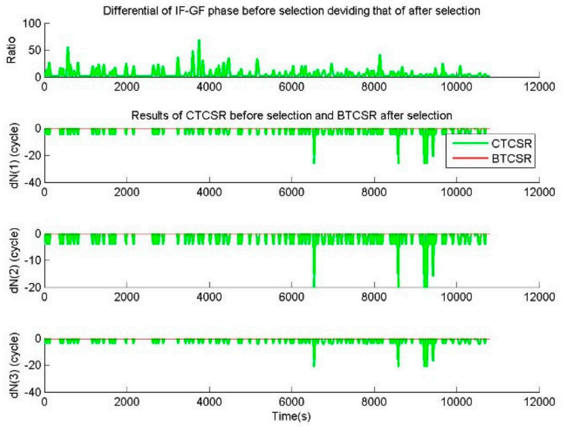

Figure S11. The cycle slip values provided from BTCSR and CTCSR to the MEO satellite 11 in the case of pseudorange data with additional errors of 1.5 m and phase data without cycle slip. Where the BDS data was collected by CUT0 at south latitude of 32 degrees, started from UTC 2016/05/08 00:00:00.

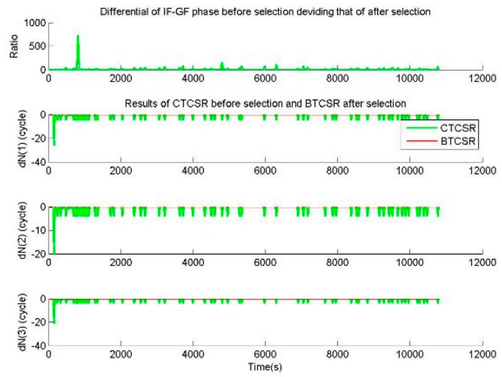

Figure S12. The cycle slip values provided from BTCSR and CTCSR to the MEO satellite 14 in the case of pseudorange data with additional errors of 1.5 m and phase data without cycle slip. Where the BDS data was collected by CUT0 at south latitude of 32 degrees, started from UTC 2016/05/08 00:00:00.

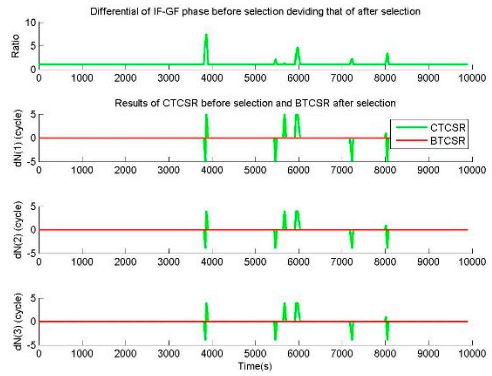

Figure S13. The cycle slip values provided from BTCSR and CTCSR to the IGSO satellite 7 in the case of normal pseudorange data and phase data without cycle slip. Where the BDS data was collected by CUT0 at south latitude of 32 degrees, started from UTC 2016/05/08 00:00:00.

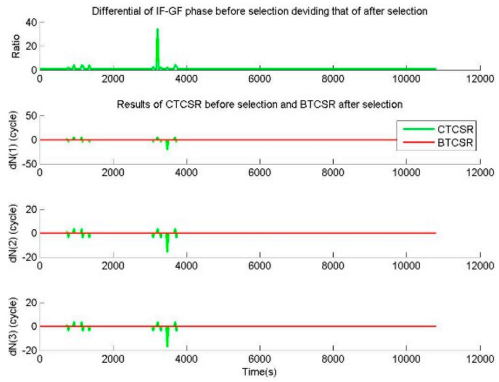

Figure S14. The cycle slip values provided from BTCSR and CTCSR to the IGSO satellite 9 in the case of normal pseudorange data and phase data without cycle slip. Where the BDS data was collected by CUT0 at south latitude of 32 degrees, started from UTC 2016/05/08 00:00:00.

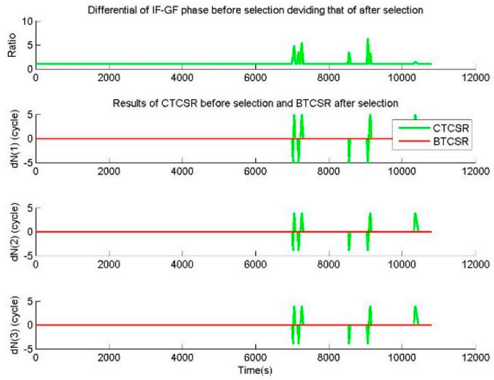

Figure S15. The cycle slip values provided from BTCSR and CTCSR to the IGSO satellite 10 in the case of normal pseudorange data and phase data without cycle slip. Where the BDS data was collected by CUT0 at south latitude of 32 degrees, started from UTC 2016/05/08 00:00:00.

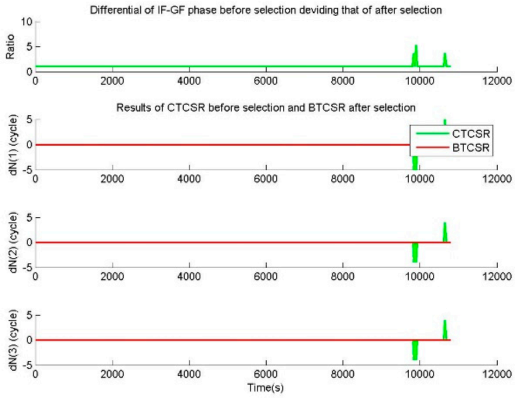

Figure S16. The cycle slip values provided from BTCSR and CTCSR to the MEO satellite 11 in the case of normal pseudorange data and phase data without cycle slip. Where the BDS data was collected by CUT0 at south latitude of 32 degrees, started from UTC 2016/05/08 00:00:00.

2. The Repaired Performances with Different Methods from GEO/IGSO/MEO Satellites

In the case of epoch-difference pseudoranges with additional 1.5 m errors and phases without cycle slip, the comparisons before and after cycle slip corrections with BTCSR and CTCSR are shown in Figures S17–S28. In the case of normal pseudorange data and phase data with slips of (1,1,1) cycles on (B1,B2,B3), the comparisons before and after cycle slip corrections with BTCSR and CTCSR are shown in Figures S29–S32 For other cycle slips corrections, the results are similar.

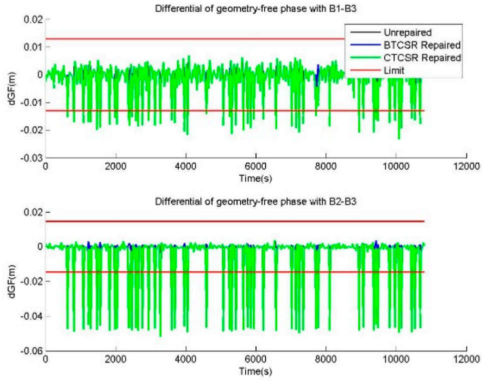

Figure S17. The differentials of geometry-free phases provided by the unrepaired data and the repaired data with BTCSR and CTCSR in the case of epoch-difference pseudoranges with additional 1.5 m errors and phases without cycle slip, where the observation data was collected from GEO satellite 1 by CUT0 at south latitude of 32 degrees started from UTC 2016/05/08 00:00:00.

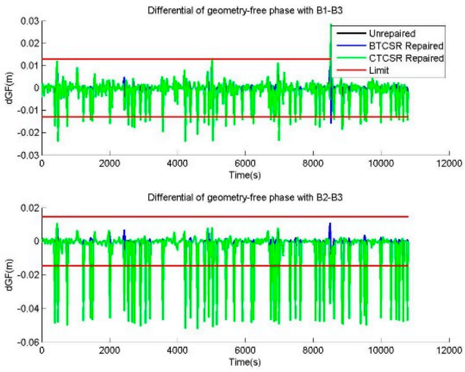

Figure S18. The differentials of geometry-free phases provided by the unrepaired data and the repaired data with BTCSR and CTCSR in the case of epoch-difference pseudoranges with additional 1.5 m errors and phases without cycle slip, where the observation data was collected from GEO satellite 2 by CUT0 at south latitude of 32 degrees started from UTC 2016/05/08 00:00:00.

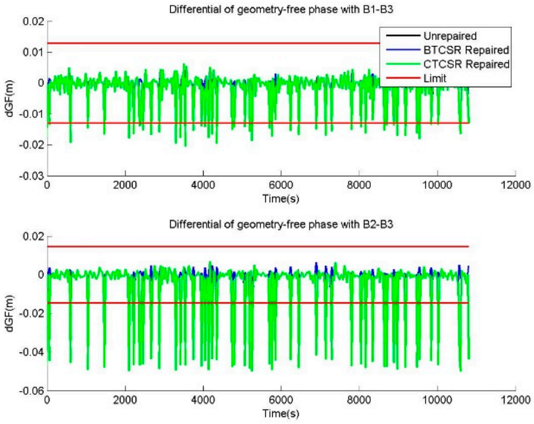

Figure S19. The differentials of geometry-free phases provided by the unrepaired data and the repaired data with BTCSR and CTCSR in the case of epoch-difference pseudoranges with additional 1.5 m errors and phases without cycle slip, where the observation data was collected from GEO satellite 3 by CUT0 at south latitude of 32 degrees started from UTC 2016/05/08 00:00:00.

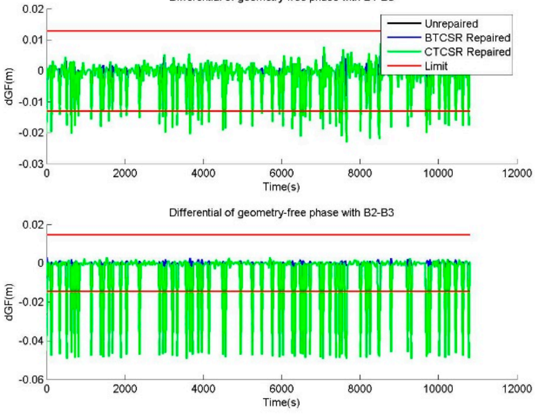

Figure S20. The differentials of geometry-free phases provided by the unrepaired data and the repaired data with BTCSR and CTCSR in the case of epoch-difference pseudoranges with additional 1.5 m errors and phases without cycle slip, where the observation data was collected from GEO satellite 4 by CUT0 at south latitude of 32 degrees started from UTC 2016/05/08 00:00:00.

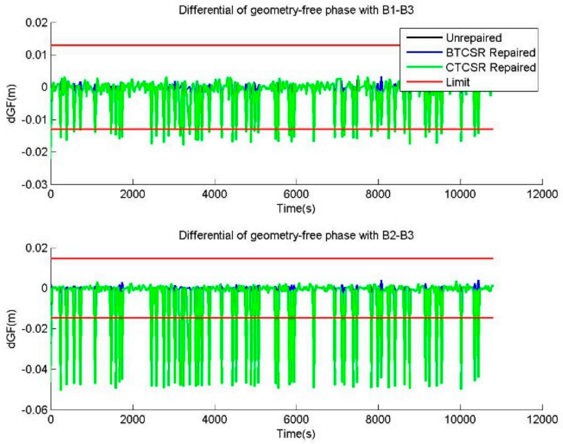

Figure S21. The differentials of geometry-free phases provided by the unrepaired data and the repaired data with BTCSR and CTCSR in the case of epoch-difference pseudoranges with additional 1.5 m errors and phases without cycle slip, where the observation data was collected from GEO satellite 5 by CUT0 at south latitude of 32 degrees started from UTC 2016/05/08 00:00:00.

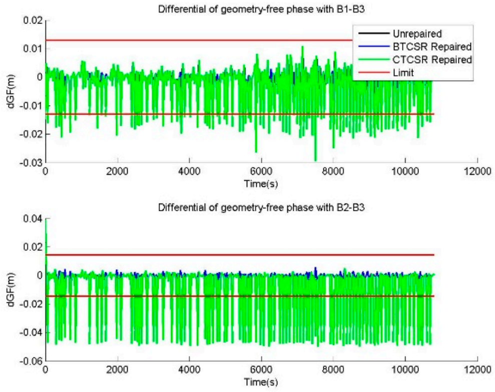

Figure S22. The differentials of geometry-free phases provided by the unrepaired data and the repaired data with BTCSR and CTCSR in the case of epoch-difference pseudoranges with additional 1.5 m errors and phases without cycle slip, where the observation data was collected from IGSO satellite 6 by CUT0 at south latitude of 32 degrees started from UTC 2016/05/08 00:00:00.

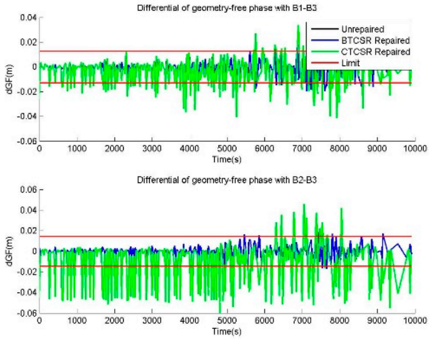

Figure S23. The differentials of geometry-free phases provided by the unrepaired data and the repaired data with BTCSR and CTCSR in the case of epoch-difference pseudoranges with additional

1.5 m errors and phases without cycle slip, where the observation data was collected from IGSO satellite 7 by CUT0 at south latitude of 32 degrees started from UTC 2016/05/08 00:00:00.

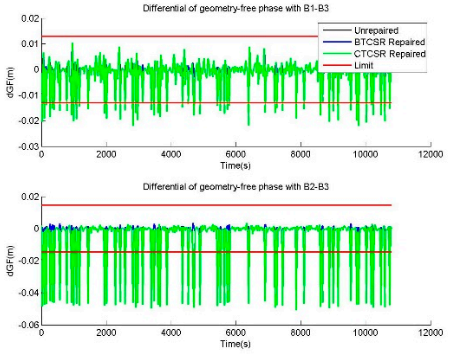

Figure S24. The differentials of geometry-free phases provided by the unrepaired data and the repaired data with BTCSR and CTCSR in the case of epoch-difference pseudoranges with additional 1.5 m errors and phases without cycle slip, where the observation data was collected from IGSO satellite 8 by CUT0 at south latitude of 32 degrees started from UTC 2016/05/08 00:00:00.

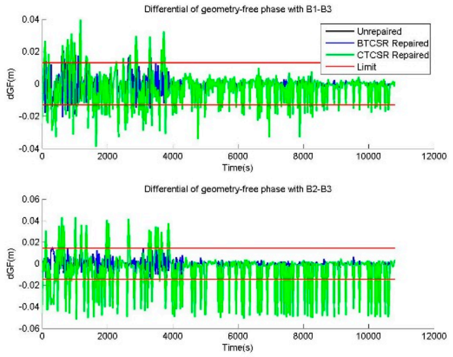

Figure S25. The differentials of geometry-free phases provided by the unrepaired data and the repaired data with BTCSR and CTCSR in the case of epoch-difference pseudoranges with additional 1.5 m errors and phases without cycle slip, where the observation data was collected from IGSO satellite 9 by CUT0 at south latitude of 32 degrees started from UTC 2016/05/08 00:00:00.

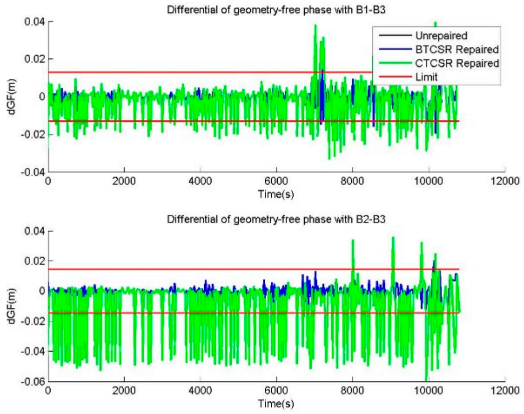

Figure S26. The differentials of geometry-free phases provided by the unrepaired data and the repaired data with BTCSR and CTCSR in the case of epoch-difference pseudoranges with additional 1.5 m errors and phases without cycle slip, where the observation data was collected from IGSO satellite 10 by CUT0 at south latitude of 32 degrees started from UTC 2016/05/08 00:00:00.

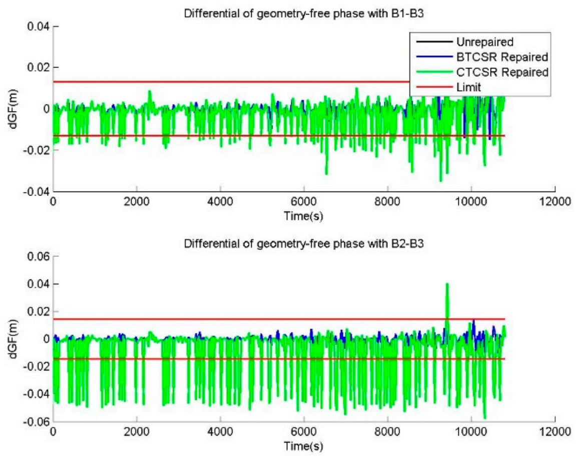

Figure S27. The differentials of geometry-free phases provided by the unrepaired data and the repaired data with BTCSR and CTCSR in the case of epoch-difference pseudoranges with additional 1.5 m errors and phases without cycle slip, where the observation data was collected from MEO satellite 11 by CUT0 at south latitude of 32 degrees started from UTC 2016/05/08 00:00:00.

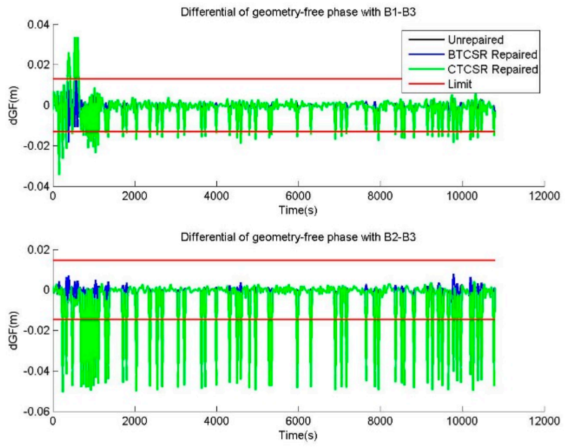

Figure S28. The differentials of geometry-free phases provided by the unrepaired data and the repaired data with BTCSR and CTCSR in the case of epoch-difference pseudoranges with additional 1.5 m errors and phases without cycle slip, where the observation data was collected from MEO satellite 14 by CUT0 at south latitude of 32 degrees started from UTC 2016/05/08 00:00:00.

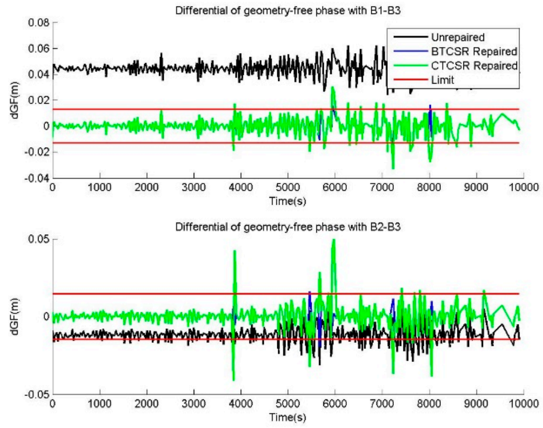

Figure S29. The differentials of geometry-free phases provided by the unrepaired data and the repaired data with BTCSR and CTCSR in the case of normal pseudorange data and phase data with

slips of (1,1,1) cycles on (B1,B2,B3), where the observation data was collected from IGSO satellite 7 by CUT0 at south latitude of 32 degrees started from UTC 2016/05/08 00:00:00.

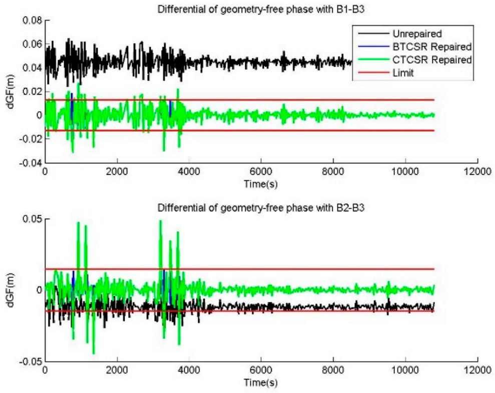

Figure S30. The differentials of geometry-free phases provided by the unrepaired data and the repaired data with BTCSR and CTCSR in the case of normal pseudorange data and phase data with slips of (1,1,1) cycles on (B1,B2,B3), where the observation data was collected from IGSO satellite 9 by CUT0 at south latitude of 32 degrees started from UTC 2016/05/08 00:00:00.

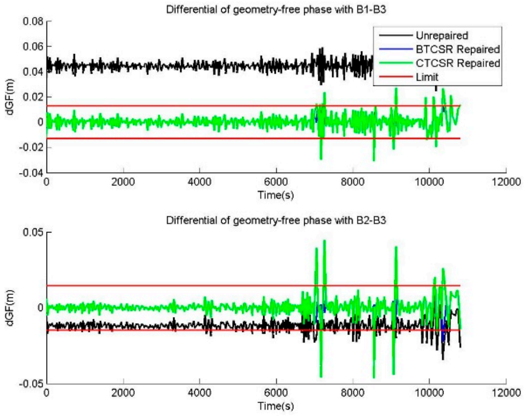

Figure S31. The differentials of geometry-free phases provided by the unrepaired data and the repaired data with BTCSR and CTCSR in the case of normal pseudorange data and phase data with slips of (1,1,1) cycles on (B1,B2,B3), where the observation data was collected from IGSO satellite 10 by CUT0 at south latitude of 32 degrees started from UTC 2016/05/08 00:00:00.

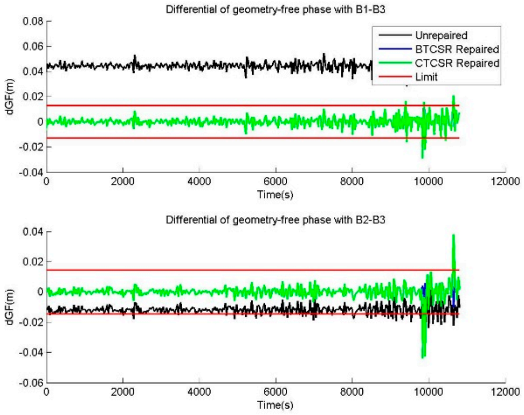

Figure S32. The differentials of geometry-free phases provided by the unrepaired data and the repaired data with BTCSR and CTCSR in the case of normal pseudorange data and phase data with

slips of (1,1,1) cycles on (B1,B2,B3), where the observation data was collected from MEO satellite 11 by CUT0 at south latitude of 32 degrees started from UTC 2016/05/08 00:00:00.
